# Characterizing health-related quality of life and identifying disease predictors among patients suspected of having long COVID: an analysis of COMET-ICE clinical trial data

**DOI:** 10.3389/fpubh.2024.1278106

**Published:** 2024-05-09

**Authors:** Heather L. Gelhorn, Parima Ghafoori, Katelyn Cutts, Helen Birch, Yulia Savva, Sacha Satram, Emily Lloyd, Wen-Hung Chen

**Affiliations:** ^1^Evidera, Bethesda, MD, United States; ^2^GSK, Collegeville, PA, United States; ^3^GSK, Brentford, United Kingdom; ^4^Vir Biotechnology, Inc., San Francisco, CA, United States

**Keywords:** COVID-19, long COVID, health-related quality of life, impacts, disease predictor, post-acute sequelae of COVID-19

## Abstract

**Introduction:**

Long COVID affects health-related quality of life (HRQoL). Here, we investigate the extent to which symptoms experienced during the acute phase of COVID-19 are significant predictors of the presence of long COVID at 12 weeks.

**Methods:**

Post-hoc analysis of COMET-ICE trial data, which assessed sotrovimab vs. placebo for treatment of mild-to-moderate COVID-19 among high-risk patients. Patient-reported outcome measures were completed during the trial, including the inFLUenza Patient-Reported Outcome Plus (FLU-PRO Plus), the 12-Item Short Form (SF-12) Hybrid questionnaire, and the Work Productivity and Activity Impairment Questionnaire: General Health (WPAI:GH). COVID-19 symptoms and impacts (measured by the FLU-PRO Plus) and HRQoL (measured by SF-12 Hybrid and WPAI:GH) were compared between the acute phase (Days 1–21 and 29) and long-COVID phase (at Week 12) among patients with and without long COVID based on COMET-ICE data. Subgroups experiencing long COVID were derived using “All,” “Returning,” and “Persisting” symptomatic definitions. Long-COVID predictors were identified using a multivariate logistic regression model; odds ratios (ORs) and 95% CIs were calculated.

**Results:**

Long-COVID subgroups had significantly higher baseline scores for most FLU-PRO Plus domains and Total Score compared with the non-long-COVID group. WPAI:GH and SF-12 Hybrid scores generally showed significantly more impairment for the long-COVID subgroups at baseline and Week 12 vs. the non-long-COVID group. In the univariate analyses, all FLU-PRO Plus domains were significant predictors of long COVID (all *p <* 0.05), with the exception of the Sense domain. Older age increased the risk of long COVID (OR 1.02, 95% CI 1.00–1.04, *p <* 0.05). Non-White patients were significantly less likely to have long COVID by the Returning and Persisting definitions vs. White patients (all *p <* 0.05). In the multivariate analysis, higher scores for the Nose domain (ORs 3.39–5.60, all *p <* 0.01) and having COPD (ORs 3.75–6.34, all *p <* 0.05) were significant long-COVID predictors.

**Conclusion:**

Patients who progressed to long COVID had higher symptom severity during the acute disease phase and showed significantly greater negative impact on HRQoL over an extended time period from initial infection through at least the subsequent 3 months. The FLU-PRO Plus Nose domain and having COPD were significant predictors of long COVID.

## 1 Introduction

In December 2019, an outbreak of novel severe acute respiratory syndrome coronavirus-2, causing the serious respiratory illness coronavirus disease 2019 (COVID-19), occurred (1). According to the World Health Organization, as of October 2023, around 770 million confirmed COVID-19 cases and around 6.9 million confirmed deaths have been reported worldwide (2), with approximately 660 million cases known to have recovered (3).

Despite the high chance of recovery from acute COVID-19, patients may experience prolonged symptoms after initial onset of disease; these people are described as having long COVID or post-COVID-19 conditions (4). Up to an estimated 80% of COVID-19 infections are associated with long-term symptoms (5–7), which can include fatigue, shortness of breath, chest pain, cough, joint and muscle pain, palpitations, headaches, “brain fog,” loss of or impaired taste and smell, lack of appetite, sore throat, diarrhea, difficulty sleeping, and depression (8–11). As well as the high symptom burden, there is increasing recognition that long COVID has a substantial negative impact on both mental and physical components of health-related quality of life (HRQoL) (12–15).

There is no exact definition of long COVID; however, some of the recent evolving definitions suggest a persistence of symptoms beyond a few weeks from acute onset of COVID-19 symptoms which is not explained by an alternative diagnosis [4 weeks according to the Centers for Disease Control and Prevention (4), 12 weeks according to the National Institute for Health and Care Excellence (16), and 3 months according to the World Health Organization (17)]. Based on a literature review (18), there are at least two descriptions of long COVID: (1) ongoing symptomatic COVID-19, which is present from 4 to 12 weeks beyond the acute phase, and (2) chronic or long COVID, which is when symptoms persist beyond 12 weeks of the onset of the acute phase of COVID-19 and are not attributable to other diagnoses (19, 20).

The dual-action monoclonal antibody sotrovimab was developed to treat mild-to-moderate COVID-19 and was shown in the COMET-ICE clinical trial (NCT04545060) to significantly reduce the absolute relative risk of >24-h hospitalization or death due to any cause by 79% vs. placebo among patients at high risk of progression to severe disease (21).

At the time this study was conducted, it was unclear if any of the cardinal symptoms of COVID-19 were predictive of the occurrence of long COVID. The current study used data from the COMET-ICE trial (conducted August 2020 to March 2021) to investigate the extent to which symptoms experienced during the acute phase of COVID-19 are significant predictors of the presence of long COVID at 12 weeks.

## 2 Materials and methods

### 2.1 Study design

This was a post-hoc analysis of data from the randomized, double-blind, multicenter, placebo-controlled, phase II/III COMET-ICE trial of sotrovimab (VIR-7831-5001) (21). The primary objective of this analysis was to evaluate differences in characteristics for patients with and without long COVID. The relationship between the acute phase of COVID-19 (Days 1–29) and the long-COVID-19 phase (after Day 29, assessed at Week 12) in terms of symptoms and impacts, as captured by the inFLUenza Patient-Reported Outcome Plus (FLU-PRO Plus) was assessed. Key patient characteristics such as age, sex, body mass index (BMI), and hospitalization status were also assessed. HRQoL, as captured by the 12-Item Short Form (SF-12) Hybrid questionnaire and the Work Productivity and Activity Impairment Questionnaire: General Health (WPAI:GH), was compared between those with and without long COVID, as defined in this study using FLU-PRO Plus data, during the acute and follow-up phases. As an exploratory objective, symptoms experienced during the acute phase of COVID-19 were also assessed by week (Weeks 1–3).

In COMET-ICE, patients with early, mild-to-moderate COVID-19 who were at high risk of progression to severe disease were randomized 1:1 to receive a single 500 mg intravenous infusion of either sotrovimab or equal volume saline placebo. Eligible patients were aged ≥18 years, had tested positive for severe acute respiratory syndrome coronavirus-2 by reverse transcription polymerase chain reaction or antigen test, had oxygen saturation of ≥94% on room air, and had symptom onset within the previous 5 days. Patients had to be considered at high risk for COVID-19 progression to hospitalization or death, as identified by the following risk factors: aged ≥55 years, or having diabetes requiring medication, obesity, chronic kidney disease, congestive heart failure, chronic obstructive pulmonary disease (COPD), or moderate/severe asthma. Patients were treated in a blinded manner with a single intravenous dose on Day 1 and followed up to 24 weeks (21). Patients were not eligible for the trial if they were currently hospitalized or judged to require hospitalization in the next 24 h, or likely to die in the next 7 days.

This analysis of COMET-ICE data did not compare the treatment groups (sotrovimab vs. placebo) for risk of long COVID.

### 2.2 Measures

Table 1 indicates the study stage, visit week and visit day when patients in the COMET-ICE trial completed the various patient-reported outcome (PRO) measures.

**Table 1:** Assessment schedule of PRO measures up to Week 12 in the COMET-ICE trial.

Study stage	Screening	Assessments				Follow-up period		
Study visit week		W1		W2	W3	W4	W8	W12
Study visit D ± visit window		D1	D8 ± 1	D15 ± 1	D22 ± 1	D29 ± 2	D57 ± 4	D85 ± 7
FLU-PRO Plus		X (daily through D21)				X	X	X
Vital signs (including oxygen saturation)	X	X	X	X	X	X		
WPAI:GH questionnaire		X		X		X	X	X
SF-12 Hybrid questionnaire		X		X		X	X	X

D, Day; FLU-PRO Plus, inFLUenza Patient-Reported Outcome Plus; PRO, patient-reported outcome; SF-12, 12-Item Short Form; W, Week; WPAI:GH, Work Productivity and Activity Impairment: General Health.

The COVID-19-adapted FLU-PRO Plus symptom questionnaire contains the 32 original FLU-PRO items assessed over the past 24 h, plus two COVID-19-specific items (loss of taste and loss of smell making up the Sense domain). The 34 FLU-PRO Plus items are scored across seven domains: Nose (four items), Throat (three items), Eyes (three items), Chest/Respiratory (seven items), Gastrointestinal (four items), Body/Systemic (11 items), and Sense (two items), primarily on a 5-point severity/frequency scale (from “Not at all” to “Very much” or “Never” to “Always,” depending on the item) (22–24). Higher scores (both total and for the individual domains and items) indicate more severe symptoms. In the COMET-ICE trial, patients were asked to complete the FLU-PRO Plus daily through Day 21, as well as at Week 4 (Day 29), Week 8, and Week 12.

The SF-12 Hybrid v2.0 is an assessment used to measure aspects of physical and mental health over the past 24 h (25). The questionnaire contains eight domains, including Physical Functioning, Role Physical, Bodily Pain, General Health, Vitality, Social Functioning, Role Emotional, and Mental Health. Two summary scores are reported from the SF-12, a Mental Component Summary score and a Physical Component Summary score. Scores are transformed into a z-score for comparison to normative data. In the normative data, the mean score is set to 50: scores >50 indicate better physical or mental health than the mean, and scores <50 indicate worse physical or mental health than the mean.

The WPAI:GH v2.0 is a 6-item questionnaire to assess absenteeism (work time missed), presenteeism (reduced on-the-job effectiveness), work productivity loss, and activity impairment due to a patient’s health condition (26). The scoring of this instrument is expressed as impairment percentages, with higher numbers indicating greater impairment and less productivity.

The WPAI:GH and SF-12 Hybrid were completed at baseline and Weeks 2, 4, 8, and 12.

Clinical and sociodemographic characteristics for patients in the COMET-ICE trial were evaluated by clinician report (hospitalization status, comorbidities, BMI, number of COVID-19 risk factors, and oxygen supplementation) and patient report (age, sex, race, and smoking and alcohol use).

### 2.3 Analysis

#### 2.3.1 Long-COVID group identifiers

**Figure 1:**
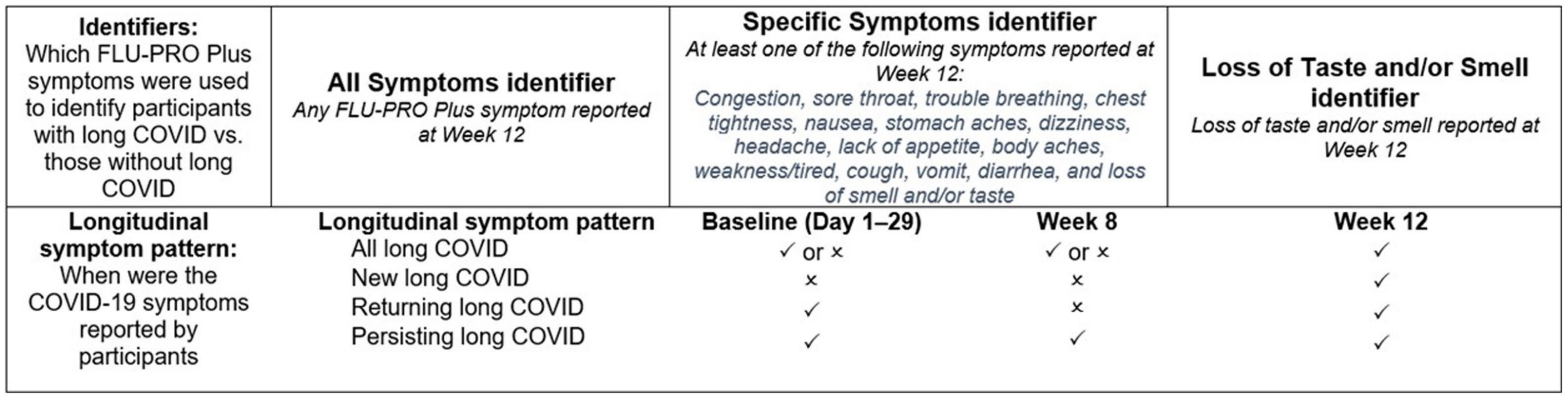
Long-COVID symptom identifiers and timepoints of symptom presence. 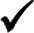 indicates symptom was present at this timepoint; 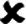 indicates symptom was not reported at this timepoint. COVID, coronavirus disease 2019; FLU-PRO Plus, inFLUenza Patient-Reported Outcome Plus.

In order to conduct the analyses, it was first necessary to derive groups representing patients with and without long COVID (Figure 1). As there is no standard definition of long COVID, several potential definitions were explored based on the results of a targeted review of the literature and consultation amongst the study team.

Three long-COVID symptom group identifiers were defined *a priori*, based on data at Week 12: (“All Symptoms,” “Specific Symptoms,” and “Loss of Smell/Taste”). The “All Symptoms” group identification was based on all FLU-PRO Plus symptoms. Any participant with 1 or more FLU-PRO Plus symptom at Week 12 was classified as long COVID under this definition. The “Specific Symptoms” definition was based on Centers for Disease Control and Prevention, National Institute for Health and Care Excellence, and World Health Organization guidelines for identifying cases of long COVID. Participants who reported specific long-COVID characteristic symptoms at Week 12 were classified as having long COVID (4, 12, 13). These symptoms included: congestion, sore throat, trouble breathing, chest tightness, nausea, stomach aches, dizziness, headache, lack of appetite, body aches, weakness/tired, cough, vomit, diarrhea, and loss of smell or taste. Finally, the third long-COVID symptom group was the “Loss of Smell/Taste” group, and considered only those who reported having lost their sense of smell and/or taste at Week 12 as long-COVID cases. For each analysis, the non-long-COVID reference group was defined as all other patients in the COMET-ICE trial who had FLU-PRO Plus data but did not fall into the long-COVID groups as defined above.

In addition to classifying participants as having long COVID or not using the three group identifiers above, four longitudinal subgroups were defined *a priori* to examine different patterns of symptom experience over time. This was intended to explore the extent to which some patients consistently experienced the same COVID symptoms over the 12-week period, or alternatively experienced different symptoms at Week 12 than they had reported during the acute phase.

- “All” (if symptom [s] had been reported at Week 12, irrespective of previous reporting).
- “Persisting” (if the symptom [s] had been reported at Week 12 and at Week 8, and was reported ≥3 times from Days 1–21 and Day 29).
- “New” (if the symptom [s] had been reported at Week 12 but not from Days 1–21 or Day 29).
- “Returning” (if the symptom [s] had been reported at Week 12 but not at Week 8, and was reported ≥3 times from Days 1–21 or Day 29).

#### 2.3.2 Descriptive statistics

Descriptive statistics (number, frequency, mean, median, interquartile range (Q1–Q3), standard deviation, and ranges) were used to summarize baseline demographic and clinical characteristics of the different long-COVID and non-long-COVID groups. In an exploratory analysis, HRQoL and FLU-PRO Plus scores were compared between long-COVID and non-long-COVID groups at baseline and Week 12, controlling for COPD status at baseline, and COPD status and baseline PRO score at Week 12.

#### 2.3.3 Regression analyses

All analyses were performed in SAS Enterprise Guide 7.15 software. A staged approach was used to explore acute COVID-19 factors associated with long-COVID cases. The binary outcome for each analysis was long-COVID case status based on the “All Symptoms” and “Specific Symptoms” identifiers described in Figure 1. The variables (i.e., predictors) included in the univariate models included FLU-PRO Plus items and domains during the acute phase (Days 1–29), clinical and demographic characteristics (age, sex, race, BMI, and hospitalization status), and comorbidities (diabetes, chronic kidney disease, moderate/severe asthma, and COPD).

Symptoms, as defined by the FLU-PRO Plus, were summarized by symptom presence, worst symptom severity score (worst symptom severity level from Days 1–21 and Day 29), average symptom severity (average symptom severity/score level from Days 1–21 and Day 29), frequency symptom score (sum of days the symptom had been reported from Days 1–21 and Day 29 divided by the number of non-missing days), and a composite symptom score (average of worst symptom severity score and frequency symptom score scaled to 0–1).

The stepwise analysis process first involved including each of the predictors measured during the acute phase (Days 1–29) in univariate logistic regressions to evaluate each as an independent predictor of the binary long COVID status at Week 12. This information was used to identify potentially relevant variables for a subsequent multivariate logistic regression. Predictors with *p <* 0.05 were considered for the next step. C-index (area under the curve [AUC]), as a measure of the classification performance, was also considered. The AUC thresholds were interpreted as follows: <0.5 no discrimination, 0.7–0.8 is acceptable, 0.8–0.9 is excellent, >0.9 is outstanding (27). For the purposes of this exploratory analysis, preliminary thresholds of 0.625 and 0.650 were used to help visualize the results across the analyses for all FLU-PRO Plus items and domains. This information was used to inform subsequent steps in the analysis. The association between these selected factors and the binary outcome was characterized by odds ratios (ORs) with 95% confidence intervals (CIs). A multivariate logistic regression model predicting the odds of being classified as having long COVID was then created based on the results from the prior step. The final multivariate models for all three long-COVID subgroups included all the FLU-PRO Plus domains as well as age, sex, race, BMI, and COPD. In the final model, the ORs were estimated and the associated 95% CIs were calculated.

## 3 Results

The results for the “All Symptoms” and the “Specific Symptoms” long-COVID identifiers were highly consistent across all analyses; results for the “All Symptoms” identifier are therefore reported below (“Specific Symptoms” results reported in the Supplementary material, Appendix 1). Results for the Loss of Smell/Taste identifier and the New Symptoms subgroup are not reported due to inadequate sample sizes.

### 3.1 Sample characteristics

The mean age of patients in the three long-COVID subgroups (All, Returning, and Persisting) was consistent and ranged from 54.3 to 54.8 years; the mean age among patients without long COVID was younger at 50.3 years (Table 2). The proportion of male patients was 42.9% in the All subgroup, lowest in the Returning subgroup at 34.2%, 42.5% in the Persisting subgroup, and 44.5% in the non-long-COVID group.

**Table 2:** Patient characteristics during the acute period (Days 1–21 and 29) for “All Symptoms” long-COVID group.

	Non-long COVID (*n* = 247)	Long COVID		
		All (*n* = 289)	Returning (*n* = 146)	Persisting (*n* = 160)
**Age, years**				
Mean (SD)	50.3 (15.4)	54.6 (12.6)	54.3 (12.6)	54.8 (12.6)
Median [Q1–Q3]	52.0 [39.0–60.0]	56.0 [47.0–63.0]	55.5 [47.0–63.0]	57.0 [46.0–64.0]
Range	18.0–94.0	21.0–86.0	21.0–86.0	21.0–82.0
**Sex, *n* (%)**				
Male	110 (44.5)	124 (42.9)	50 (34.2)	68 (42.5)
Female	137 (55.5)	165 (57.1)	96 (65.8)	92 (57.5)
**Race, *n* (%)**				
White	193 (78.1)	251 (87.2)	133 (91.1)	148 (92.5)
Black or African American	31 (12.6)	27 (9.4)	8 (5.5)	10 (6.3)
Asian	22 (8.9)	8 (2.8)	4 (2.7)	2 (1.3)
American Indian or Alaska Native	0 (0.0)	1 (0.3)	1 (0.7)	0 (0.0)
Multiple	1 (0.4)	1 (0.3)	0 (0.0)	0 (0.0)
**BMI**				
Mean (SD)	32.2 (6.6)	32.7 (7.2)	32.7 (6.3)	32.8 (7.4)
Median [Q1–Q3]	31.7 [27.5–35.6]	32.3 [27.8–35.9]	32.8 [28.1–35.9]	32.3 [27.6–36.0]
Range	18.7–60.5	17.7–71.2	17.7–53.5	17.7–71.1
**Medical conditions, *n* (%)**				
Obesity	158 (64.0)	191 (66.1)	100 (68.5)	105 (65.6)
Diabetes	55 (22.3)	69 (23.9)	29 (19.9)	41 (25.6)
Chronic kidney disease	4 (1.6)	1 (0.3)	1 (0.7)	1 (0.6)
Congestive heart failure	0 (0.0)	2 (0.7)	0 (0.0)	0 (0.0)
COPD	5 (2.0)	24 (8.3)	12 (8.2)	17 (10.6)
Moderate/severe asthma	43 (17.4)	41 (14.2)	24 (16.4)	29 (18.1)
**Number of risk factors, *n* (%)**^a^				
0	2 (0.8)	0 (0.0)	0 (0.0)	0 (0.0)
1	142 (57.5)	147 (50.9)	74 (50.7)	68 (42.5)
2	77 (31.2)	95 (32.9)	49 (33.6)	63 (39.4)
≥3	26 (10.5)	47 (16.3)	23 (15.8)	29 (18.1)
**Hospitalization status, *n* (%)**^b^				
Hospitalization and/or ER visit and/or death	7 (2.8)	13 (4.5)	7 (4.8)	7 (4.4)
**Oxygen supplementation, *n* (%)**^c^				
Room air	247 (100.0)	288 (99.7)	146 (100.0)	160 (100.0)
Other	0 (0.0)	1 (0.3)	0 (0.0)	0 (0.0)

For proportions, only non-missing values were included in denominator.

a Risk factors: age ≥ 55 years, diabetes requiring medication, obesity (BMI >30 kg/m^2^), chronic kidney disease (estimated glomerular filtration rate < 60 mL/min/1.73 m^2^ by modification of diet in renal disease), congestive heart failure (New York Heart Association class II or more), COPD, moderate/severe asthma, total number of conditions.

b At Days 1–21 and Day 29.

c At baseline.

BMI, body mass index; COPD, chronic obstructive pulmonary disease; COVID, coronavirus disease 2019; ER, emergency room; SD, standard deviation.

In the long-COVID subgroups, the proportions of patients who were White ranged from 87.2 to 92.5%, the proportions who were Black or African American ranged from 5.5 to 9.4%, and the proportions who were Asian ranged from 1.3 to 2.8%. In the non-long-COVID group, these proportions were 78.1, 12.6, and 8.9%, respectively.

Only 2.0% of patients in the non-long-COVID group reported COPD, compared with 8.2–10.6% of patients in the long-COVID subgroups (Table 2). Other comorbidities were relatively consistent across the groups. The proportion of patients with ≥3 risk factors was 15.8–18.1% among the long-COVID subgroups and 10.5% in the non-long-COVID group. Hospitalization status was 4.4–4.8% among the long-COVID subgroups and 2.8% in the non-long-COVID group.

### 3.2 HRQoL at baseline and Week 12

Baseline PRO scores differed for some scales on the WPAI:V and D SF-12 Hybrid between non-long-COVID and long-COVID groups, even when adjusted for baseline COPD status (Table 3). At baseline, patients with long-COVID by the All and Returning definitions had significantly greater activity impairment than those without long-COVID based on the WPAI:GH (*p <* 0.05). However, overall work impairment, impairment while working, and time missed from work did not differ significantly between groups at baseline. For the SF-12 Hybrid, Role Physical and Vitality domain scores were significantly lower (all *p <* 0.05) for all long-COVID subgroups compared with non-long-COVID (with the exception of Role Physical in the Persisting subgroup).

**Table 3:** WPAI:GH and SF-12 Hybrid scores at baseline, “All Symptoms” long-COVID group, adjusted for baseline COPD status.

PRO items	Non-long COVID n Mean (SE) *n =* 247	All long COVID n Mean (SE) *n =* 289	Returning long COVID n Mean (SE) *n =* 146	Persisting long COVID n Mean (SE) *n =* 160
**WPAI:GH**				
Activity impairment due to health	112 52.0 (2.9)	**146** **59.8 (2.6)**	**76** **62.6 (3.6)**	92 59.7 (3.2)
Overall work impairment due to health	33 52.4 (5.6)	57 63.8 (4.3)	26 57.2 (6.3)	38 65.9 (5.2)
Impairment while working due to health	33 39.5 (5.5)	58 47.8 (4.1)	26 45.4 (6.2)	38 48.4 (5.1)
Work time missed due to health	102 55.7 (3.9)	135 50.8 (3.4)	62 47.5 (5.0)	80 48.7 (4.4)
**SF-12 Hybrid**				
Role Physical domain	219 55.2 (1.8)	**262** **47.9 (1.7)**	**132** **45.9 (2.3)**	145 50.3 (2.2)
Vitality domain	219 38.9 (1.9)	**262** **32.2 (1.7)**	**132** **32.4 (2.5)**	**145** **30.8 (2.3)**
General Health domain	219 56.6 (1.9)	262 54.1 (1.7)	132 56.4 (2.4)	145 55.2 (2.3)
Mental Component Summary score	219 43.4 (0.7)	262 42.4 (0.7)	132 41.4 (0.9)	145 42.3 (0.9)
Physical Component Summary score	219 44.5 (0.6)	**262** **42.6 (0.5)**	132 42.7 (0.7)	145 43.2 (0.7)

Bold cells indicate a significant difference in mean PRO value at baseline relative to the non-long-COVID group at alpha <0.05. COPD, chronic obstructive pulmonary disease; COVID, coronavirus disease 2019; PRO, patient-reported outcome.

SE, standard error; SF-12, 12-Item Short Form; WPAI:GH, Work Productivity and Activity Impairment Questionnaire: General Health.

There were also differences between the long-COVID and non-long-COVID groups at Week 12, even when adjusting for COPD status and baseline PRO score. At Week 12, patients with long COVID had significantly greater impairment while working, overall work impairment, and activity impairment (all *p <* 0.05) based on the WPAI:GH (Table 4). Time missed from work did not differ significantly between the non-long-COVID and long-COVID groups. For the SF-12 Hybrid, all domain and component scores were significantly lower compared with those with long COVID (all *p <* 0.05), suggesting worse functioning and health among those with long COVID.

**Table 4:** WPAI:GH and SF-12 Hybrid scores at Week 12, “All Symptoms” long-COVID group, adjusted for baseline COPD status and PRO score^a^.

PRO items	Non-long COVID n Mean (SE) *n =* 247	All long COVID n Mean (SE) *n =* 289	Returning long COVID n Mean (SE) *n =* 146	Persisting long COVID n Mean (SE) *n =* 160
**WPAI:GH**				
Activity impairment due to health	208 14.8 (2.7)	**221** **34.3 (2.5)**	**107** **37.5 (3.6)**	**119** **34.9 (3.2)**
Overall work impairment due to health	91 11.0 (5.1)	**123** **25.3 (3.5)**	**57** **33.3 (5.3)**	**67** **25.3 (4.2)**
Impairment while working due to health	91 8.7 (4.7)	**123** **22.8 (3.2)**	**57** **29.7 (4.9)**	**67** **23.4 (3.9)**
Work time missed due to health	93 4.7 (2.2)	128 6.3 (1.9)	59 6.9 (2.8)	70 5.9 (2.4)
**SF-12 Hybrid**				
Role Physical domain	215 89.5 (1.7)	**235** **73.1 (1.6)**	**115** **70.2 (2.3)**	**129** **69.0 (2.2)**
Vitality domain	215 72.3 (2.0)	**234** **51.5 (1.9)**	**115** **49.5 (2.8)**	**129** **48.8 (2.6)**
General Health domain	215 77.8 (1.5)	**233** **65.7 (1.5)**	**114** **64.3 (2.1)**	**127** **62.4 (2.0)**
Mental Component Summary score	215 54.7 (0.6)	**234** **49.5 (0.6)**	**115** **48.2 (0.9)**	**129** **48.7 (0.8)**
Physical Component Summary score	215 54.4 (0.5)	**234** **49.7 (0.5)**	**115** **49.3 (0.7)**	**129** **48.7 (0.7)**

Bold cells indicate a significant difference in mean PRO value at baseline relative to the non-long-COVID group at alpha <0.05.

a Each PRO domain is adjusted for baseline score of the specific value. COPD, chronic obstructive pulmonary disease; COVID, coronavirus disease 2019; PRO, patient-reported outcome; SE, standard error; SF-12, 12-Item Short Form; WPAI:GH, Work Productivity and Activity Impairment Questionnaire: General Health.

### 3.3 FLU-PRO Plus scores

**Figure 2:**
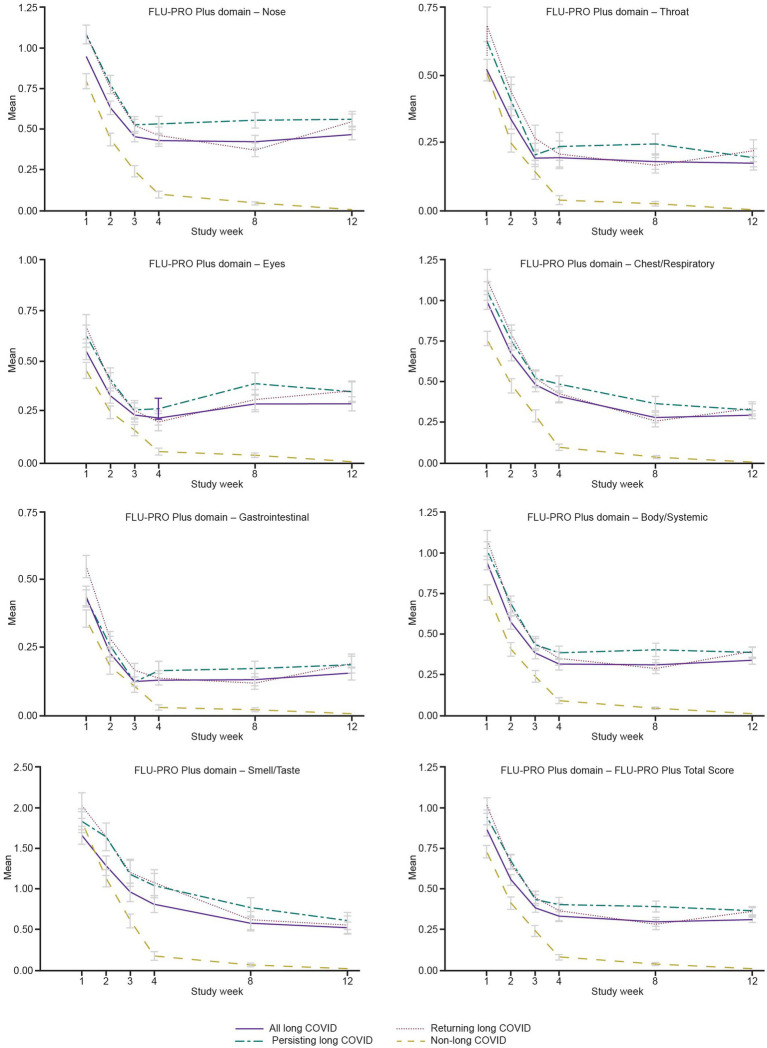
Mean FLU-PRO Plus domain scores from Weeks 1 to 12, “All Symptoms” long-COVID group. COVID, coronavirus disease 2019; FLU-PRO Plus, inFLUenza Patient-Reported Outcome Plus.

The “All Symptoms” long-COVID subgroup showed significantly more severe baseline scores for all domains and the Total Score (all *p <* 0.05; adjusted for baseline COPD status) compared with the non-long-COVID group, with the exception of the Throat domain. Baseline scores for the Nose, Eyes, Gastrointestinal, and Body/Systemic domains were significantly more severe in all three long-COVID subgroups (all *p <* 0.05) compared with the non-long-COVID group (Table 5 and Figure 2).

**Table 5:** FLU-PRO Plus scores at baseline, “All Symptoms” long-COVID group, adjusted for baseline COPD status.

PRO items	Non-long COVID n Mean (SE) *n =* 247	All long COVID n Mean (SE) *n =* 289	Returning long COVID n Mean (SE) *n =* 146	Persisting long COVID n Mean (SE) *n =* 160
**FLU-PRO Plus**				
Nose	247 1.2 (0.1)	**289** **1.4 (0.1)**	**146** **1.6 (0.1)**	**160** **1.5 (0.1)**
Throat	247 0.9 (0.1)	289 1.0 (0.1)	146 1.1 (0.1)	160 1.1 (0.1)
Eyes	247 0.8 (0.1)	**289** **0.9 (0.1)**	**146** **1.0 (0.1)**	**160** **1.0 (0.1)**
Chest/Respiratory	247 1.2 (0.1)	**289** **1.3 (0.0)**	**146** **1.4 (0.1)**	160 1.3 (0.1)
Gastrointestinal	247 0.6 (0.0)	**289** **0.8 (0.0)**	**146** **0.9 (0.1)**	**160** **0.8 (0.1)**
Body/Systemic	247 1.4 (0.1)	**289** **1.6 (0.0)**	**146** **1.6 (0.1)**	**160** **1.5 (0.1)**
Sense	247 2.2 (0.1)	**289** **1.8 (0.1)**	146 2.0 (0.2)	**160** **1.7 (0.1)**
FLU-PRO Plus Total Score	247 1.2 (0.0)	**289** **1.3 (0.0)**	**146** **1.4 (0.1)**	160 1.3 (0.1)

Bold cells indicate a significant difference in mean PRO value at baseline relative to the non-long-COVID group at alpha <0.05. COPD, chronic obstructive pulmonary disease; COVID, coronavirus disease 2019; FLU-PRO Plus, inFLUenza Patient-Reported Outcome Plus; PRO, patient-reported outcome; SE, standard error.

### 3.4 Univariate long-COVID prediction

A heatmap of the results of the univariate analyses is presented in Table 6. The results tables for each of the average severity analyses are provided in the Supplementary material (Appendix 2, Supplementary Tables 1, 2).

**Table 6:** Heatmap of the AUC results from the univariate analyses of individual FLU-PRO Plus domains and items during acute phase (Days 1–29) of COVID-19 as predictors of long-COVID status at Week 12.

FLU-PRO Plus Symptoms and Domains During Acute Phase (Days 1–29)	Average severity		Symptom presence		Worst severity		Symptom frequency		Composite score	
	All	Specific	All	Specific	All	Specific	All	Specific	All	Specific
**Nose domain**										
All long COVID	0.665	0.661								
Returning long COVID	0.720	0.726								
Persisting long COVID	0.725	0.742								
**1. Runny or dripping nose**										
All long COVID	0.624	0.616	0.587	0.572	0.601	0.595	0.635	0.623	0.631	0.621
Returning long COVID	0.676	0.685	0.637	0.644	0.650	0.664	0.684	0.690	0.690	0.698
Persisting long COVID	0.663	0.666	0.623	0.618	0.626	0.623	0.678	0.680	0.673	0.671
**2. Congested or stuffy nose**										
All long COVID	0.625	0.619	0.587	0.571	0.576	0.571	0.645	0.634	0.625	0.618
Returning long COVID	0.673	0.678	0.612	0.604	0.619	0.628	0.688	0.684	0.672	0.678
Persisting long COVID	0.688	0.701	0.635	0.629	0.610	0.621	0.718	0.727	0.688	0.698
**3. Sinus pressure**										
All long COVID	0.603	0.600	0.571	0.563	0.572	0.564	0.617	0.613	0.609	0.602
Returning long COVID	0.668	0.676	0.620	0.619	0.618	0.625	0.685	0.686	0.675	0.685
Persisting long COVID	0.657	0.676	0.625	0.627	0.596	0.610	0.683	0.700	0.659	0.677
**28. Sneezing**										
All long COVID	0.657	0.666	0.599	0.581	0.537	0.543	0.703	0.702	0.657	0.662
Returning long COVID	0.701	0.701	0.630	0.611	0.567	0.582	0.745	0.729	0.700	0.699
Persisting long COVID	0.702	0.722	0.630	0.608	0.531	0.541	0.766	0.775	0.699	0.714
**Throat domain**										
All long COVID	0.564	0.565								
Returning long COVID	0.624	0.622								
Persisting long COVID	0.592	0.614								
**4. Scratchy or itchy throat**										
All long COVID	0.587	0.586	0.541	0.541	0.578	0.567	0.588	0.588	0.596	0.590
Returning long COVID	0.641	0.631	0.587	0.589	0.621	0.613	0.644	0.633	0.648	0.639
Persisting long COVID	0.620	0.634	0.577	0.578	0.600	0.595	0.627	0.644	0.624	0.631
**5. Sore or painful throat**										
All long COVID	0.545	0.547	0.509	0.502	0.523	0.521	0.557	0.558	0.548	0.548
Returning long COVID	0.599	0.604	0.543	0.545	0.565	0.570	0.615	0.618	0.599	0.603
Persisting long COVID	0.568	0.596	0.529	0.547	0.527	0.543	0.587	0.615	0.563	0.587
**6. Difficulty swallowing**										
All long COVID	0.517	0.515	0.511	0.517	0.521	0.514	0.518	0.514	0.519	0.515
Returning long COVID	0.582	0.584	0.540	0.529	0.577	0.578	0.584	0.587	0.582	0.583
Persisting long COVID	0.529	0.549	0.508	0.531	0.522	0.533	0.533	0.553	0.528	0.546
**Eyes domain**										
All long COVID	0.599	0.592								
Returning long COVID	0.655	0.652								
Persisting long COVID	0.642	0.650								
**7. Teary or watery eyes**										
All long COVID	0.577	0.576	0.548	0.528	0.577	0.570	0.581	0.579	0.580	0.579
Returning long COVID	0.634	0.628	0.602	0.575	0.621	0.617	0.639	0.631	0.636	0.628
Persisting long COVID	0.612	0.634	0.588	0.590	0.597	0.613	0.623	0.644	0.615	0.637
**8. Sore or painful eyes**										
All long COVID	0.582	0.567	0.565	0.537	0.570	0.553	0.585	0.568	0.582	0.566
Returning long COVID	0.624	0.623	0.606	0.581	0.600	0.598	0.628	0.623	0.619	0.619
Persisting long COVID	0.610	0.607	0.597	0.582	0.585	0.583	0.616	0.614	0.606	0.606
**9. Eyes sensitive to light**										
All long COVID	0.561	0.562	0.549	0.538	0.554	0.556	0.565	0.564	0.564	0.564
Returning long COVID	0.605	0.609	0.594	0.573	0.584	0.598	0.608	0.606	0.607	0.612
Persisting long COVID	0.595	0.605	0.582	0.579	0.566	0.569	0.602	0.612	0.594	0.602
**Chest/Respiratory domain**										
All long COVID	0.652	0.679								
Returning long COVID	0.700	0.704								
Persisting long COVID	0.681	0.732								
**10. Trouble breathing**										
All long COVID	0.589	0.613	0.554	0.563	0.572	0.592	0.594	0.616	0.591	0.615
Returning long COVID	0.655	0.659	0.595	0.587	0.624	0.627	0.661	0.663	0.654	0.658
Persisting long COVID	0.623	0.678	0.585	0.625	0.594	0.643	0.632	0.686	0.625	0.681
**11. Chest congestion**										
All long COVID	0.610	0.629	0.581	0.595	0.579	0.593	0.614	0.631	0.611	0.628
Returning long COVID	0.672	0.686	0.632	0.652	0.630	0.629	0.677	0.693	0.673	0.684
Persisting long COVID	0.638	0.682	0.610	0.635	0.593	0.623	0.650	0.693	0.638	0.679
**12. Chest tightness**										
All long COVID	0.581	0.591	0.564	0.581	0.554	0.559	0.589	0.596	0.581	0.588
Returning long COVID	0.639	0.630	0.616	0.614	0.596	0.587	0.647	0.637	0.635	0.626
Persisting long COVID	0.615	0.657	0.609	0.648	0.565	0.599	0.631	0.670	0.611	0.653
**13. Dry or hacking cough**										
All long COVID	0.595	0.618	0.554	0.566	0.563	0.573	0.612	0.633	0.605	0.621
Returning long COVID	0.630	0.637	0.569	0.570	0.573	0.576	0.644	0.649	0.631	0.632
Persisting long COVID	0.618	0.663	0.565	0.587	0.573	0.599	0.642	0.685	0.628	0.665
**14. Wet or loose cough**										
All long COVID	0.608	0.627	0.556	0.562	0.560	0.573	0.623	0.639	0.606	0.622
Returning long COVID	0.630	0.642	0.577	0.582	0.586	0.605	0.641	0.649	0.630	0.641
Persisting long COVID	0.630	0.656	0.575	0.585	0.575	0.580	0.649	0.674	0.630	0.648
**29. Coughing**										
All long COVID	0.656	0.686	0.570	0.574	0.562	0.580	0.694	0.716	0.667	0.692
Returning long COVID	0.699	0.705	0.592	0.581	0.588	0.604	0.734	0.728	0.707	0.714
Persisting long COVID	0.686	0.749	0.594	0.601	0.561	0.599	0.738	0.793	0.695	0.751
**30. Coughed up mucus or phlegm**										
All long COVID	0.611	0.632	0.564	0.565	0.551	0.575	0.636	0.649	0.614	0.634
Returning long COVID	0.636	0.640	0.588	0.571	0.564	0.587	0.663	0.657	0.639	0.645
Persisting long COVID	0.636	0.664	0.596	0.597	0.552	0.574	0.674	0.693	0.640	0.664
**Gastrointestinal domain**										
All long COVID	0.626	0.640								
Returning long COVID	0.693	0.699								
Persisting long COVID	0.635	0.665								
**15. Felt nauseous**										
All long COVID	0.549	0.560	0.536	0.548	0.546	0.553	0.550	0.561	0.551	0.560
Returning long COVID	0.588	0.602	0.596	0.604	0.570	0.587	0.594	0.608	0.587	0.600
Persisting long COVID	0.543	0.570	0.544	0.565	0.538	0.565	0.547	0.573	0.543	0.570
**16. Stomach ache**										
All long COVID	0.572	0.579	0.557	0.550	0.546	0.554	0.585	0.589	0.569	0.575
Returning long COVID	0.642	0.637	0.633	0.619	0.602	0.593	0.657	0.653	0.638	0.631
Persisting long COVID	0.583	0.598	0.569	0.569	0.548	0.566	0.601	0.610	0.576	0.591
**31. How many times did you vomit?**										
All long COVID	0.534	0.538	0.507	0.507	0.531	0.534	0.534	0.539	0.532	0.535
Returning long COVID	0.551	0.553	0.516	0.516	0.549	0.551	0.551	0.554	0.549	0.551
Persisting long COVID	0.532	0.545	0.516	0.522	0.528	0.540	0.532	0.545	0.530	0.542
**32. How many times did you have diarrhea?**										
All long COVID	0.649	0.661	0.594	0.593	0.624	0.633	0.649	0.660	0.641	0.652
Returning long COVID	0.709	0.723	0.654	0.660	0.667	0.683	0.708	0.721	0.697	0.711
Persisting long COVID	0.660	0.684	0.617	0.626	0.631	0.646	0.665	0.684	0.654	0.673
**Body/Systemic domain**										
All long COVID	0.639	0.664								
Returning long COVID	0.706	0.723								
Persisting long COVID	0.686	0.739								
**17. Felt dizzy**										
All long COVID	0.582	0.589	0.564	0.556	0.556	0.559	0.595	0.597	0.583	0.590
Returning long COVID	0.613	0.613	0.597	0.586	0.583	0.585	0.625	0.617	0.613	0.614
Persisting long COVID	0.618	0.645	0.603	0.609	0.579	0.602	0.635	0.655	0.617	0.647
**18. Head congestion**										
All long COVID	0.611	0.621	0.566	0.556	0.587	0.596	0.624	0.629	0.621	0.628
Returning long COVID	0.676	0.701	0.604	0.605	0.631	0.659	0.688	0.705	0.685	0.712
Persisting long COVID	0.686	0.710	0.612	0.608	0.637	0.658	0.706	0.726	0.699	0.720
**19. Headache**										
All long COVID	0.613	0.620	0.589	0.581	0.556	0.564	0.638	0.641	0.613	0.620
Returning long COVID	0.664	0.670	0.626	0.618	0.576	0.587	0.694	0.695	0.656	0.664
Persisting long COVID	0.663	0.694	0.620	0.624	0.579	0.599	0.699	0.725	0.662	0.692
**20. Lack of appetite**										
All long COVID	0.555	0.583	0.566	0.575	0.544	0.583	0.568	0.588	0.567	0.597
Returning long COVID	0.618	0.634	0.612	0.624	0.587	0.612	0.633	0.641	0.629	0.644
Persisting long COVID	0.579	0.618	0.590	0.600	0.551	0.601	0.601	0.630	0.592	0.634
**21. Sleeping more than usual**										
All long COVID	0.580	0.595	0.536	0.531	0.560	0.559	0.597	0.608	0.592	0.597
Returning long COVID	0.606	0.609	0.553	0.549	0.575	0.561	0.622	0.619	0.614	0.607
Persisting long COVID	0.584	0.616	0.542	0.549	0.542	0.544	0.612	0.642	0.594	0.612
**22. Body aches or pains**										
All long COVID	0.609	0.639	0.576	0.578	0.555	0.570	0.642	0.667	0.620	0.642
Returning long COVID	0.667	0.684	0.613	0.610	0.593	0.597	0.705	0.717	0.673	0.682
Persisting long COVID	0.654	0.711	0.612	0.634	0.575	0.608	0.699	0.756	0.665	0.716
**23. Weak or tired**										
All long COVID	0.649	0.679	0.567	0.566	0.577	0.601	0.692	0.710	0.666	0.689
Returning long COVID	0.710	0.721	0.612	0.604	0.616	0.629	0.756	0.751	0.729	0.732
Persisting long COVID	0.710	0.774	0.607	0.619	0.595	0.647	0.769	0.822	0.725	0.785
**24. Chills or shivering**										
All long COVID	0.535	0.550	0.524	0.525	0.532	0.544	0.550	0.562	0.539	0.554
Returning long COVID	0.572	0.578	0.572	0.570	0.543	0.547	0.596	0.596	0.570	0.576
Persisting long COVID	0.540	0.561	0.539	0.552	0.526	0.537	0.563	0.587	0.542	0.561
**25. Felt cold**										
All long COVID	0.598	0.605	0.586	0.575	0.576	0.582	0.605	0.606	0.601	0.608
Returning long COVID	0.628	0.630	0.613	0.598	0.588	0.593	0.639	0.634	0.625	0.631
Persisting long COVID	0.621	0.640	0.622	0.632	0.581	0.589	0.641	0.659	0.619	0.635
**26. Felt hot**										
All long COVID	0.573	0.579	0.558	0.556	0.560	0.564	0.574	0.578	0.577	0.581
Returning long COVID	0.639	0.650	0.613	0.627	0.604	0.611	0.642	0.653	0.637	0.645
Persisting long COVID	0.571	0.590	0.571	0.579	0.546	0.567	0.577	0.591	0.572	0.593
**27. Sweating**										
All long COVID	0.551	0.558	0.526	0.530	0.542	0.540	0.557	0.565	0.563	0.564
Returning long COVID	0.613	0.616	0.580	0.591	0.583	0.574	0.628	0.629	0.621	0.619
Persisting long COVID	0.564	0.592	0.555	0.577	0.527	0.542	0.585	0.612	0.571	0.593
**Sense domain**										
All long COVID	0.503	0.537								
Returning long COVID	0.572	0.620								
Persisting long COVID	0.552	0.601								
**33. Loss of smell**										
All long COVID	0.503	0.535	0.536	0.512	0.522	0.512	0.503	0.535	0.503	0.535
Returning long COVID	0.568	0.613	0.518	0.560	0.505	0.535	0.568	0.613	0.568	0.613
Persisting long COVID	0.547	0.595	0.503	0.542	0.512	0.504	0.547	0.595	0.547	0.595
**34. Loss of taste**										
All long COVID	0.507	0.542	0.513	0.511	0.528	0.510	0.507	0.542	0.507	0.542
Returning long COVID	0.569	0.614	0.542	0.577	0.506	0.526	0.569	0.614	0.569	0.614
Persisting long COVID	0.550	0.596	0.523	0.557	0.524	0.501	0.550	0.596	0.550	0.596
**FLU-PRO Plus Total Score**										
All long COVID	0.652	0.676								
Returning long COVID	0.723	0.742								
Persisting long COVID	0.702	0.756								

(“All Symptoms” long-COVID group). Analyses that resulted in AUC ≥0.65 are shaded in dark green and AUC ≥0.625 are shaded in light green. Unshaded areas represent AUC values <0.625.

AUC, area under the curve; COVID-19, coronavirus disease 2019; FLU-PRO Plus, inFLUenza Patient-Reported Outcome Plus.

Overall, the univariate analyses suggest that significant predictor variables were most often able to identify long COVID among the Returning and Persisting subgroups. In general, the average symptom severity, symptom frequency, and composite scores were better predictors of long COVID, while symptom presence and worst symptom severity scores were less useful as predictors.

All FLU-PRO Plus domains were significant predictors of long COVID using both the “All Symptoms” and “Specific Symptoms” identifiers (all *p <* 0.05), with the exception of Sense (scored as a binary Yes/No domain) for the All long-COVID subgroup for the “All Symptoms” long-COVID identifier. Domains that were the most predictive based on the univariate regression models (AUCs >0.625) for the All, Returning, and Persisting long-COVID subgroups, respectively, were Nose (AUCs 0.67, 0.72, and 0.73), Chest/Respiratory (AUCs 0.65, 0.70, and 0.68), Gastrointestinal (AUCs 0.63, 0.69, and 0.64), and Body/Systemic (AUCs 0.64, 0.71, and 0.69). The FLU-PRO Plus Total Score was a significant predictor of long COVID for all long-COVID subgroups (all *p <* 0.05; AUCs 0.65, 0.72, and 0.70). Almost all FLU-PRO Plus symptoms during the acute period (Days 1–21 and Day 29) were significant predictors of long COVID; the items that most consistently showed the greatest predictive power (AUCs >0.65) included “runny or dripping nose,” “congested or stuffy nose,” “sinus pressure,” “sneezing,” “trouble breathing,” “chest congestion,” “coughing,” “coughed up mucus or phlegm,” “frequency of diarrhea,” “head congestion,” “headache,” “body aches or pains,” and “weak or tired,” and were therefore clustered within the Nose, Chest/Respiratory and Body/Systemic domains.

The following demographic and clinical characteristics were included in univariate analyses to evaluate if they were significant predictors of any of the long-COVID groups: age, sex, race (White, Black, or Other), BMI, diabetes, chronic kidney disease, moderate/severe asthma, COPD, and hospitalization status. Older age was associated with an increased risk of long COVID (OR 1.02, 95% CI 1.00–1.04, all *p <* 0.05). Race was a significant predictor for each of the long-COVID groups. Minority patients (Black and of other races) were significantly less likely to have long COVID by the Returning definition (ORs 0.37 and 0.32, both *p <* 0.05) and Persisting definition (ORs 0.42 and 0.11, both *p <* 0.05) compared with White patients. Those classified as “Other” race were also significantly less likely to have All long COVID (OR 0.33, 95% CI 0.16–0.72, *p* = 0.01) compared with White patients. COPD status was the strongest predictor of long COVID for all three groups (OR 4.33–5.75, all *p <* 0.01).

### 3.5 Multivariate long-COVID prediction

As each of the FLU-PRO Plus domains were predictive of long COVID, the final multivariate models for all three long-COVID subgroups included the FLU-PRO Plus domains as well as significant and/or relevant covariates such as age, sex, race, BMI, and COPD status.

Results were generally consistent across all three long-COVID subgroups (Table 7 – All long COVID; Appendix 2, Supplementary Table 3 – Returning long COVID; Appendix 2, Supplementary Table 4 – Persisting long COVID). In these multivariate analyses, higher scores on the Nose domain (OR 3.39–5.60, all *p <* 0.01) and having COPD (OR 3.75–6.34, all *p <* 0.05) were significant predictors of having long COVID, with COPD being the strongest overall predictor. Increasing age was also a significant risk factor for long COVID (OR 1.03–1.04, all *p <* 0.001). Patients reporting “Other” race were less likely to have long COVID by the All and Persisting definitions compared with White patients (ORs 0.40 and 0.16, *p <* 0.05). Higher scores on the Throat domain were a significant negative predictor for the All long COVID subgroup, but not for the Returning or Persisting subgroups (OR 0.47, *p <* 0.05).

**Table 7:** Final model of long-COVID predictors for “All Symptoms” long-COVID group.

Parameters	B (SE)	*p*-value	Wald Chi-square	OR	95% CI for OR	
					Lower	Upper
**FLU-PRO Plus domains**						
Nose	1.22 (0.34)	0.0003	12.94	3.39	1.74	6.59
Throat	−0.76 (0.34)	0.0239	5.10	0.47	0.24	0.90
Eyes	−0.20 (0.33)	0.5374	0.38	0.81	0.43	1.56
Chest/Respiratory	0.54 (0.29)	0.0631	3.45	1.72	0.97	3.04
Gastrointestinal	0.59 (0.47)	0.2078	1.59	1.80	0.72	4.50
Body/Systemic	0.20 (0.37)	0.5892	0.29	1.22	0.60	2.49
Sense	−0.13 (0.08)	0.1144	2.49	0.88	0.75	1.03
**Demographic and clinical characteristics**						
Age, years	0.03 (0.01)	<0.0001	18.55	1.03	1.02	1.05
Sex, Ref: Female	0.08 (0.19)	0.6926	0.16	1.08	0.74	1.58
Black/African American race, Ref: White	−0.19 (0.30)	0.5414	0.37	0.83	0.46	1.51
Other race, Ref: White	−0.92 (0.42)	0.0266	4.92	0.40	0.18	0.90
BMI, kg/m^2^	0.02 (0.01)	0.1933	1.69	1.02	0.99	1.05
COPD	1.47 (0.53)	0.0059	7.59	4.34	1.53	12.34
**Model fit**						
Number of observations	535					
AIC	677.15					
-2Log likelihood	649.15					
R^2^, Cox-Snell, Max-rescaled	0.1538, 0.2055					
Score, Chi-square (DF, *p*-value)	80.92 (13, <0.0001)					
Wald Chi-square (DF, *p*-value)	68.59 (13, <0.0001)					
Hosmer-Lemeshow, Chi-square (DF, *p*-value)	9.72 (8, 0.2855)					
C-index (AUC)	0.7229					

AIC, Akaike information criterion; AUC, area under the curve; BMI, body mass index; CI, confidence interval; COPD, chronic obstructive pulmonary disease; COVID, coronavirus disease 2019; DF, degree of freedom; FLU-PRO Plus, inFLUenza Patient-Reported Outcome Plus; OR, odds ratio; SE, standard error.

## 4 Discussion

This study used an existing clinical trial dataset and *a priori*-constructed symptom-based definitions of long COVID to retrospectively explore the HRQoL burden of long COVID, as well as identify significant predictors of long COVID. Our results suggest that patients who progress to long COVID have significantly greater impacts of the disease even during the acute phase (Days 1–21 and 29), both in terms of some aspects of HRQoL and their symptom severity.

Previous studies have identified several demographic and clinical characteristics that might be predictive of long COVID, including female sex, advancing age, the number of symptoms during the acute phase of the disease, and the severity of symptoms (28–31). However, prior to conducting the analyses, it was unknown whether any key symptom(s) might emerge as harbingers of long COVID. The results of the current study suggest that a large number of individual symptoms during the acute phase are significant but relatively weak individual predictors of long COVID. This is perhaps not surprising given the high degree of heterogeneity in the COVID-19 symptom experience that is observed, both in these data and in the wider literature on the general and long-COVID symptom experience.

When examined in a multivariate context, higher scores on the Nose and Chest/Respiratory domains emerged as predictive of long COVID, as did several sociodemographic and clinical characteristics, including age, race, and COPD. One of the most notable findings of the current investigation is, in fact, not that any individual symptoms are noteworthy or particularly powerful predictors of long COVID, but rather that the severity of the symptoms and the disease overall during the acute phase seems to be consistently related to the subsequent emergence of long COVID. This finding is in agreement with previous studies (28, 29). Patients who progress to long COVID appear to already be more severely affected at the onset and during the acute phase of the disease, while presenting with very heterogeneous symptom presentations. Nevertheless, others have shown that long COVID can also occur in a substantial proportion (20 to 30%) of individuals after mild disease.

We identified a relatively high number of patients in the long-COVID subgroups who had COPD [8.2–10.6%, compared with an age-adjusted prevalence of 5.6% in the United States in 2020], which was higher than in the non-long-COVID subgroup (2.0%). We also found that COPD was predictive of long COVID, which is consistent with the results of another larger study of non-hospitalized adults on predictors of long COVID, which found that many comorbid conditions were predictive of the persistence of the disease, with COPD being the most strongly related (32). However, findings related to age and race differ from those of the prior study, possibly due to notable differences in the makeup of the samples for each study. While the current study is based on a clinical trial sample with an older average age and patients at high risk of disease progression, the prior study was a broader population-based study. It should be noted that our sample size of patients with COPD was limited, and further research is required on COPD as a predictor of long COVID.

### 4.1 Strengths and limitations

Some strengths of the current study include that patients reported their symptoms in an electronic daily diary during the acute phase of the disease (Days 1–21), the same measure was used to gather these symptom data during multiple follow-up evaluations, and the initial COVID-19 diagnoses were laboratory-confirmed.

The study also has some limitations which should be considered. Firstly, this study is based on a clinical trial population of generally older patients who were deemed to have a high risk of progression to severe COVID-19; our results may therefore not be representative of long COVID among the general population. Data on symptoms and HRQoL before patients had COVID-19 (and were included in the COMET-ICE trial) were not available; hence there is a risk of confounding by underlying disease. For example, reported symptoms may have been due to conditions such as COPD or other chronic conditions, rather than long COVID. There is also a risk of social desirability bias, given that the data in the study was self-reported by the patients. As this was a post-hoc analysis, we were also not able to verify that patients included in these analyses had long COVID as data were blinded; our assumption was based on available FLU-PRO Plus PRO data from the COMET-ICE trial and patients were not verified as having long COVID by a clinician. We also did not control the analysis for treatment arm (sotrovimab or placebo). We only assessed symptoms included in the FLU-PRO Plus, and so other symptoms that have been identified for long COVID may not have been evaluated. There were also low PRO completion rates by patients in the COMET-ICE trial. Finally, we only report data to Week 12 and are therefore unable to explore longer-term symptoms and impacts of long COVID.

## 5 Conclusion

In conclusion, the current study suggests that patients who progress to long COVID had higher symptom severity during the acute phase of the disease, and showed significant HRQoL impacts over an extended period of time from initial infection through at least the subsequent 3 months. While there were several significant but weak predictors of progression to long COVID, underlying COPD was a strong predictor that would be of interest to investigate further.

## Data availability statement

The data analyzed in this study is subject to the following licenses/restrictions: the datasets presented in this article are not readily available due to ethical and privacy concerns. Requests to access these datasets should be directed to PG, parima.s.ghafoori@gsk.com.

## Ethics statement

The studies involving humans were approved by the COMET-ICE trial (NCT04545060) independent review board. The studies were conducted in accordance with the local legislation and institutional requirements. Written informed consent for participation in this study was provided by the participants’ legal guardians/next of kin.

## Author contributions

HG: Conceptualization, Data curation, Investigation, Validation, Visualization, Writing – review & editing, Formal analysis, Methodology. PG: Conceptualization, Data curation, Investigation, Validation, Writing – review & editing, Formal analysis, Methodology, Visualization. KC: Conceptualization, Data curation, Investigation, Validation, Writing – review & editing, Formal analysis, Methodology, Visualization. HB: Conceptualization, Data curation, Investigation, Validation, Writing – review & editing. YS: Conceptualization, Data curation, Investigation, Validation, Writing – review & editing. SS: Conceptualization, Data curation, Investigation, Validation, Writing – review & editing. EL: Conceptualization, Data curation, Investigation, Validation, Writing – review & editing. W-HC: Conceptualization, Data curation, Investigation, Validation, Writing – review & editing.
