# Supplementary material for: Characterizing health-related quality of life and identifying disease predictors among patients suspected of having long COVID: an analysis of COMET-ICE clinical trial data
Source: Front Public Health. 2024 May 9;12:1278106. doi: 10.3389/fpubh.2024.1278106 (PMC11111858; doi:10.3389/fpubh.2024.1278106)
Supplement: Supplementary file 1 [file Data_Sheet_1.DOCX]

Supplementary material

**Contents**

[Appendix 1 – “Specific Symptoms” long-COVID identifier results. 2](#_Toc141193773)

[Supplementary Table 1. Patient characteristics during the acute period (Days 1–21 and 29) for “Specific Symptoms” long-COVID group. 2](#_Toc141193774)

[Supplementary Table 2. WPAI:GH and SF-12 Hybrid scores at baseline, “Specific Symptoms” long-COVID group, adjusted for baseline COPD status 4](#_Toc141193775)

[Supplementary Table 3. WPAI:GH and SF-12 Hybrid scores at Week 12, “Specific Symptoms” long-COVID group, adjusted for baseline COPD status and PRO score^a^. 5](#_Toc141193776)

[Supplementary Table 4. FLU-PRO Plus scores at baseline, “Specific Symptoms” long-COVID group, adjusted for baseline COPD status. 6](#_Toc141193777)

[Appendix 2 7](#_Toc141193778)

[Supplementary Table 1. ORs of long-COVID groups, “All Symptoms” long-COVID group: univariate models, FLU-PRO Plus items, average symptom severity.. 7](#_Toc141193779)

[Supplementary Table 2. ORs of long-COVID groups, “Specific Symptoms” long-COVID group: univariate models, FLU-PRO Plus items, average symptom severity. 11](#_Toc141193780)

[Supplementary Table 3. Final model of long-COVID predictors for “All Symptoms”, Returning long-COVID subgroup. 15](#_Toc141193781)

[Supplementary Table 4. Final model of long-COVID predictors for “All Symptoms”, Persisting long-COVID subgroup. 16](#_Toc141193782)

Appendix 1 – “Specific symptoms” long-COVID identifier results.

Supplementary Table 1. Patient characteristics during the acute period (Days 1–21 and 29) for “Specific Symptoms” long-COVID group. For proportions, only non-missing values were included in denominator. ^a^Risk factors: age ≥55 years, diabetes requiring medication, obesity (BMI >30 kg/m^2^), chronic kidney disease (estimated glomerular filtration rate
<60 mL/min/1.73m^2^ by modification of diet in renal disease), congestive heart failure (New York Heart Association class II or more), COPD, moderate/severe asthma, total number of conditions. ^b^At Days 1–21 and Day 29. ^c^At baseline. BMI, body mass index; COPD, chronic obstructive pulmonary disease; COVID, coronavirus disease 2019; ER, emergency room;
SD, standard deviation.

|  | **Non-long COVID (*n =* 286)** | **Long COVID** | | |
| --- | --- | --- | --- | --- |
|  |  | **All (*n =* 250)** | **Returning (*n =* 111)** | **Persisting (*n =* 127)** |
| **Age, years** | | | | |
| Mean (SD) | 50.7 (14.8) | 54.8 (13.0) | 53.9 (13.2) | 55.4 (12.3) |
| Median [Q1–Q3] | 52.0 [40.0–60.0] | 56.0 [47.0–64.0] | 56.0 [46.0–63.0] | 57.0 [47.0–65.0] |
| Range | 18.0–94.0 | 21.0–86.0 | 21.0–86.0 | 21.0–82.0 |
| **Sex, *n* (%)** | | | | |
| Male | 132 (46.2) | 102 (40.8) | 35 (31.5) | 53 (41.7) |
| Female | 154 (53.8) | 148 (59.2) | 76 (68.5) | 74 (58.3) |
| **Race, *n* (%)** | | | | |
| White | 228 (79.7) | 216 (86.7) | 103 (92.8) | 119 (93.7) |
| Black or African American | 33 (11.5) | 25 (10.0) | 5 (4.5) | 7 (5.5) |
| Asian | 23 (8.0) | 7 (2.8) | 3 (2.7) | 1 (0.8) |
| American Indian or  Alaska Native | 1 (0.3) | 0 (0.0) | 0 (0.0) | 0 (0.0) |
| Multiple | 1 (0.3) | 1 (0.4) | 0 (0.0) | 0 (0.0) |
| **BMI** | | | | |
| Mean (SD) | 32.3 (6.7) | 32.6 (7.1) | 32.7 (6.5) | 32.3 (7.1) |
| Median [Q1–Q3] | 32.0 [27.5–35.8] | 32.1 [27.8–35.8] | 32.9 [28.1–35.9] | 32.0 [27.6–35.7] |
| Range | 18.7–60.5 | 17.7–71.2 | 17.7–53.5 | 17.7–71.1 |
| **Medical conditions, *n* (%)** | | | | |
| Obesity | 185 (64.7) | 164 (65.6) | 76 (68.5) | 81 (63.8) |
| Diabetes | 61 (21.3) | 63 (25.2) | 22 (19.8) | 35 (27.6) |
| Chronic kidney disease | 4 (1.4) | 1 (0.4) | 1 (0.9) | 1 (0.8) |
| Congestive heart failure | 0 (0.0) | 2 (0.8) | 0 (0.0) | 0 (0.0) |
| COPD | 8 (2.8) | 21 (8.4) | 7 (6.3) | 15 (11.8) |
| Moderate/severe asthma | 48 (16.8) | 36 (14.4) | 18 (16.2) | 25 (19.7) |
| **Number of risk factors, *n* (%)^a^** | | | | |
| 0 | 2 (0.7) | 0 (0.0) | 0 (0.0) | 0 (0.0) |
| 1 | 164 (57.3) | 125 (50.0) | 59 (53.2) | 51 (40.2) |
| 2 | 88 (30.8) | 84 (33.6) | 33 (29.7) | 51 (40.2) |
| ≥3 | 32 (11.2) | 41 (16.4) | 19 (17.1) | 25 (19.7) |
| **Hospitalization status, *n* (%)^b^** | | | | |
| Hospitalization and/or ER visit and/or death | 7 (2.4) | 13 (5.2) | 6 (5.4) | 7 (5.5) |
| **Oxygen supplementation, *n* (%)^c^** | | | | |
| Room air | 286 (100.0) | 249 (99.6) | 111 (100.0) | 127 (100.0) |
| Other | 0 (0.0) | 1 (0.4) | 0 (0.0) | 0 (0.0) |

Supplementary Table 2. WPAI:GH and SF-12 Hybrid scores at baseline, “Specific Symptoms” long-COVID group, adjusted for baseline COPD status. Bold cells indicate a significant difference in mean PRO value at baseline relative to the non-long-COVID group at alpha <0.05.
COPD, chronic obstructive pulmonary disease; COVID, coronavirus disease 2019; PRO, patient-reported outcome; SE, standard error; SF-12, 12-Item Short Form; WPAI:GH, Work Productivity and Activity Impairment Questionnaire: General Health.

| **PRO items** | **Non-long COVID n**  **Mean (SE) *n =* 286** | **All long COVID n**  **Mean (SE) *n =* 250** | **Returning long COVID n**  **Mean (SE) *n =* 111** | **Persisting long COVID n**  **Mean (SE) *n =* 127** |
| --- | --- | --- | --- | --- |
| **WPAI:GH** | | | | |
| Activity impairment due to health | 133  52.9 (2.7) | 125  60.1 (2.8) | **57**  **63.9 (4.1)** | 73  61.6 (3.6) |
| Overall work impairment due to health | 43  57.5 (5.0) | 47  61.8 (4.8) | 19  60.2 (7.5) | 30  64.2 (5.9) |
| Impairment while working due to health | 43  43.6 (4.8) | 48  46.1 (4.6) | 19  45.9 (7.2) | 30  46.4 (5.8) |
| Work time missed due to health | 123  53.5 (3.6) | 114  52.2 (3.7) | 47  50.2 (5.8) | 63  47.5 (5.0) |
| **SF-12 Hybrid** | | | | |
| Role Physical domain | 253  54.9 (1.7) | **228**  **47.2 (1.8)** | **100**  **46.8 (2.7)** | **114**  **48.3 (2.5)** |
| Vitality domain | 253  38.9 (1.8) | **228**  **31.3 (1.9)** | 100  32.4 (2.8) | **114**  **29.7 (2.7)** |
| General Health domain | 253  56.7 (1.7) | 228  53.8 (1.8) | 100  57.0 (2.8) | 114  54.9 (2.6) |
| Mental Component Summary score | 253  43.7 (0.7) | 228  42.0 (0.7) | 100  41.6 (1.1) | 114  41.6 (1.0) |
| Physical Component Summary score | 253  44.4 (0.5) | **228**  **42.5 (0.6)** | 100  42.7 (0.9) | 114  42.9 (0.8) |

Supplementary Table 3. WPAI:GH and SF-12 Hybrid scores at Week 12, “Specific Symptoms” long-COVID group, adjusted for baseline COPD status and PRO score^a^. Bold cells indicate a significant difference in mean PRO value at baseline relative to the non-long-COVID group at alpha <0.05. ^a^Each PRO domain is adjusted for baseline score of the specific value.
COPD, chronic obstructive pulmonary disease; COVID, coronavirus disease 2019; PRO, patient-reported outcome; SE, standard error; SF-12, 12-Item Short Form; WPAI:GH, Work Productivity and Activity Impairment Questionnaire: General Health.

| **PRO items** | **Non-long COVID n**  **Mean (SE) *n =* 286** | **All long COVID n**  **Mean (SE) *n =* 250** | **Returning long COVID n**  **Mean (SE) *n =* 111** | **Persisting long COVID n**  **Mean (SE) *n =* 127** |
| --- | --- | --- | --- | --- |
| **WPAI:GH** | | | | |
| Activity impairment due to health | 235  15.1 (2.5) | **194**  **36.7 (2.6)** | **81**  **40.8 (4.2)** | **97**  **38.4 (3.5)** |
| Overall work impairment due to health | 107  10.3 (4.3) | **107**  **28.6 (3.8)** | **45**  **36.6 (6.2)** | **55**  **29.2 (4.7)** |
| Impairment while working due to health | 107  8.2 (4.0) | **107**  **25.8 (3.4)** | **45**  **31.7 (5.8)** | **55**  **27.5 (4.3)** |
| Work time missed due to health | 109  4.2 (2.1) | 112  6.9 (2.0) | 47  8.0 (3.2) | 58  7.0 (2.7) |
| **SF-12 Hybrid** | | | | |
| Role Physical domain | 242  89.2 (1.6) | **208**  **71.7 (1.7)** | **89**  **68.7 (2.6)** | **106**  **67.8 (2.4)** |
| Vitality domain | 242  71.0 (1.9) | **207**  **50.8 (2.1)** | **89**  **48.8 (3.2)** | **106**  **46.1 (2.9)** |
| General Health domain | 242  78.3 (1.4) | **206**  **63.8 (1.5)** | **88**  **63.7 (2.4)** | **104**  **59.8 (2.2)** |
| Mental Component Summary score | 242  54.2 (0.6) | **207**  **49.4 (0.7)** | **89**  **47.9 (1.0)** | **106**  **47.9 (0.9)** |
| Physical Component Summary score | 242  54.6 (0.5) | **207**  **49.1 (0.5)** | **89**  **48.9 (0.8)** | **106**  **48.1 (0.7)** |

Supplementary Table 4. FLU-PRO Plus scores at baseline, “Specific Symptoms” long-COVID group, adjusted for baseline COPD status. Bold cells indicate a significant difference in mean PRO value at baseline relative to the non-long-COVID group at alpha <0.05. COPD, chronic obstructive pulmonary disease; COVID, coronavirus disease 2019; FLU-PRO Plus, inFLUenza Patient-Reported Outcome Plus; PRO, patient-reported outcome; SE, standard error.

| **PRO items** | **Non-long COVID n**  **Mean (SE) *n =* 286** | **All long COVID n**  **Mean (SE) *n =* 250** | **Returning long COVID n**  **Mean (SE) *n =* 111** | **Persisting long COVID n**  **Mean (SE) *n =* 127** |
| --- | --- | --- | --- | --- |
| **FLU-PRO Plus** | | | | |
| Nose | 286  1.2 (0.1) | **250**  **1.4 (0.1)** | **111**  **1.6 (0.1)** | **127**  **1.5 (0.1)** |
| Throat | 286  0.9 (0.1) | 250  1.0 (0.1) | 111  1.1 (0.1) | 127  1.1 (0.1) |
| Eyes | 286  0.8 (0.1) | **250**  **0.9 (0.1)** | **111**  **1.1 (0.1)** | **127**  **1.0 (0.1)** |
| Chest/Respiratory | 286  1.2 (0.0) | **250**  **1.4 (0.1)** | **111**  **1.4 (0.1)** | **127**  **1.4 (0.1)** |
| Gastrointestinal | 286  0.7 (0.0) | **250**  **0.8 (0.0)** | **111**  **0.9 (0.1)** | **127**  **0.9 (0.1)** |
| Body/Systemic | 286  1.4 (0.0) | **250**  **1.6 (0.1)** | **111**  **1.6 (0.1)** | **127**  **1.6 (0.1)** |
| Sense | 286  2.1 (0.1) | 250  1.8 (0.1) | 111  2.0 (0.2) | 127  1.7 (0.2) |
| FLU-PRO Plus Total Score | 286  1.2 (0.0) | **250**  **1.3 (0.0)** | **111**  **1.4 (0.1)** | **127**  **1.4 (0.1)** |

Appendix 2

Supplementary Table 1. ORs of long-COVID groups, “All Symptoms” long-COVID group: univariate models, FLU-PRO Plus items, average symptom severity. AUC, area under the curve; CI, confidence interval; COVID, coronavirus disease 2019; FLU-PRO Plus, inFLUenza Patient-Reported Outcome Plus; OR, odds ratio; SE, standard error.

| **Parameters** | ***n*** | **B (SE)** | ***p*-value** | **Wald Chi-square** | **OR** | **95% CI for OR** | | **C-index (AUC)** |
| --- | --- | --- | --- | --- | --- | --- | --- | --- |
|  |  |  |  |  |  | **Lower** | **Upper** |  |
| **1. Runny or dripping nose** | | | | | | | | |
| All long COVID | 536 | 0.89 (0.19) | < 0.0001 | 21.50 | 2.45 | 1.68 | 3.57 | 0.624 |
| Returning long COVID | 393 | 1.22 (0.23) | < 0.0001 | 27.05 | 3.39 | 2.14 | 5.37 | 0.676 |
| Persisting long COVID | 407 | 1.15 (0.22) | < 0.0001 | 27.76 | 3.17 | 2.06 | 4.87 | 0.663 |
| **2. Congested or stuffy nose** | | | | | | | | |
| All long COVID | 536 | 0.82 (0.16) | < 0.0001 | 25.50 | 2.28 | 1.65 | 3.13 | 0.625 |
| Returning long COVID | 393 | 1.09 (0.20) | < 0.0001 | 31.32 | 2.98 | 2.03 | 4.37 | 0.673 |
| Persisting long COVID | 407 | 1.19 (0.19) | < 0.0001 | 38.74 | 3.30 | 2.26 | 4.80 | 0.688 |
| **3. Sinus pressure** | | | | | | | | |
| All long COVID | 536 | 0.76 (0.17) | < 0.0001 | 20.78 | 2.13 | 1.54 | 2.94 | 0.603 |
| Returning long COVID | 393 | 1.12 (0.20) | < 0.0001 | 30.26 | 3.07 | 2.06 | 4.57 | 0.668 |
| Persisting long COVID | 407 | 1.06 (0.19) | < 0.0001 | 31.34 | 2.88 | 1.99 | 4.16 | 0.657 |
| **4. Scratchy or itchy throat** | | | | | | | | |
| All long COVID | 536 | 0.65 (0.18) | 0.0003 | 13.34 | 1.91 | 1.35 | 2.71 | 0.587 |
| Returning long COVID | 393 | 0.94 (0.21) | < 0.0001 | 19.79 | 2.56 | 1.69 | 3.87 | 0.641 |
| Persisting long COVID | 407 | 0.84 (0.20) | < 0.0001 | 18.52 | 2.32 | 1.58 | 3.40 | 0.620 |
| **5. Sore or painful throat** | | | | | | | | |
| All long COVID | 536 | 0.38 (0.19) | 0.0402 | 4.21 | 1.46 | 1.02 | 2.11 | 0.545 |
| Returning long COVID | 393 | 0.66 (0.22) | 0.0025 | 9.18 | 1.94 | 1.26 | 2.98 | 0.599 |
| Persisting long COVID | 407 | 0.59 (0.20) | 0.0038 | 8.40 | 1.80 | 1.21 | 2.67 | 0.568 |
| **6. Difficulty swallowing** | | | | | | | | |
| All long COVID | 536 | 0.25 (0.21) | 0.2383 | 1.39 | 1.28 | 0.85 | 1.94 | 0.517 |
| Returning long COVID | 393 | 0.66 (0.26) | 0.0096 | 6.72 | 1.94 | 1.18 | 3.21 | 0.582 |
| Persisting long COVID | 407 | 0.44 (0.23) | 0.0587 | 3.57 | 1.55 | 0.98 | 2.43 | 0.529 |
| **7. Teary or watery eyes** | | | | | | | | |
| All long COVID | 536 | 0.64 (0.20) | 0.0016 | 9.99 | 1.90 | 1.28 | 2.83 | 0.577 |
| Returning long COVID | 393 | 1.03 (0.25) | < 0.0001 | 16.57 | 2.80 | 1.71 | 4.60 | 0.634 |
| Persisting long COVID | 407 | 0.93 (0.23) | < 0.0001 | 15.63 | 2.53 | 1.60 | 4.00 | 0.612 |
| **8. Sore or painful eyes** | | | | | | | | |
| All long COVID | 536 | 0.70 (0.21) | 0.0007 | 11.42 | 2.01 | 1.34 | 3.01 | 0.582 |
| Returning long COVID | 393 | 0.94 (0.25) | 0.0002 | 14.16 | 2.57 | 1.57 | 4.20 | 0.624 |
| Persisting long COVID | 407 | 0.88 (0.23) | 0.0001 | 14.47 | 2.42 | 1.54 | 3.82 | 0.610 |
| **9. Eyes sensitive to light** | | | | | | | | |
| All long COVID | 536 | 0.57 (0.18) | 0.0018 | 9.73 | 1.77 | 1.24 | 2.53 | 0.561 |
| Returning long COVID | 393 | 0.89 (0.22) | < 0.0001 | 16.64 | 2.43 | 1.59 | 3.73 | 0.605 |
| Persisting long COVID | 407 | 0.82 (0.21) | < 0.0001 | 16.02 | 2.28 | 1.52 | 3.41 | 0.595 |
| **10. Trouble breathing** | | | | | | | | |
| All long COVID | 536 | 0.74 (0.17) | < 0.0001 | 17.73 | 2.09 | 1.48 | 2.94 | 0.589 |
| Returning long COVID | 393 | 1.10 (0.21) | < 0.0001 | 27.37 | 2.99 | 1.99 | 4.52 | 0.655 |
| Persisting long COVID | 407 | 0.95 (0.20) | < 0.0001 | 22.99 | 2.60 | 1.76 | 3.84 | 0.623 |
| **11. Chest congestion** | | | | | | | | |
| All long COVID | 536 | 0.82 (0.18) | < 0.0001 | 21.37 | 2.28 | 1.61 | 3.24 | 0.610 |
| Returning long COVID | 393 | 1.17 (0.22) | < 0.0001 | 29.48 | 3.23 | 2.12 | 4.93 | 0.672 |
| Persisting long COVID | 407 | 0.98 (0.20) | < 0.0001 | 24.15 | 2.68 | 1.81 | 3.97 | 0.638 |
| **12. Chest tightness** | | | | | | | | |
| All long COVID | 536 | 0.68 (0.18) | 0.0002 | 13.66 | 1.97 | 1.38 | 2.83 | 0.581 |
| Returning long COVID | 393 | 0.99 (0.22) | < 0.0001 | 20.04 | 2.69 | 1.74 | 4.15 | 0.639 |
| Persisting long COVID | 407 | 0.87 (0.21) | < 0.0001 | 17.29 | 2.40 | 1.59 | 3.62 | 0.615 |
| **13. Dry or hacking cough** | | | | | | | | |
| All long COVID | 536 | 0.53 (0.13) | < 0.0001 | 17.10 | 1.69 | 1.32 | 2.17 | 0.595 |
| Returning long COVID | 393 | 0.67 (0.15) | < 0.0001 | 19.64 | 1.96 | 1.46 | 2.64 | 0.630 |
| Persisting long COVID | 407 | 0.63 (0.15) | < 0.0001 | 18.75 | 1.88 | 1.41 | 2.50 | 0.618 |
| **14. Wet or loose cough** | | | | | | | | |
| All long COVID | 536 | 0.73 (0.17) | < 0.0001 | 19.42 | 2.08 | 1.50 | 2.89 | 0.608 |
| Returning long COVID | 393 | 0.86 (0.20) | < 0.0001 | 18.15 | 2.37 | 1.59 | 3.52 | 0.630 |
| Persisting long COVID | 407 | 0.85 (0.19) | < 0.0001 | 19.40 | 2.34 | 1.60 | 3.42 | 0.630 |
| **15. Felt nauseous** | | | | | | | | |
| All long COVID | 536 | 0.21 (0.18) | 0.2379 | 1.39 | 1.23 | 0.87 | 1.75 | 0.549 |
| Returning long COVID | 393 | 0.33 (0.22) | 0.1338 | 2.25 | 1.39 | 0.90 | 2.13 | 0.588 |
| Persisting long COVID | 407 | 0.17 (0.20) | 0.4146 | 0.67 | 1.18 | 0.79 | 1.76 | 0.543 |
| **16. Stomach ache** | | | | | | | | |
| All long COVID | 536 | 0.51 (0.20) | 0.0114 | 6.40 | 1.67 | 1.12 | 2.47 | 0.572 |
| Returning long COVID | 393 | 0.92 (0.25) | 0.0002 | 13.80 | 2.50 | 1.54 | 4.07 | 0.642 |
| Persisting long COVID | 407 | 0.57 (0.22) | 0.0103 | 6.57 | 1.76 | 1.14 | 2.72 | 0.583 |
| **17. Felt dizzy** | | | | | | | | |
| All long COVID | 536 | 0.43 (0.16) | 0.0073 | 7.20 | 1.54 | 1.12 | 2.10 | 0.582 |
| Returning long COVID | 393 | 0.54 (0.19) | 0.0040 | 8.29 | 1.71 | 1.19 | 2.46 | 0.613 |
| Persisting long COVID | 407 | 0.60 (0.18) | 0.0007 | 11.47 | 1.82 | 1.29 | 2.58 | 0.618 |
| **18. Head congestion** | | | | | | | | |
| All long COVID | 536 | 0.73 (0.16) | < 0.0001 | 21.91 | 2.08 | 1.53 | 2.83 | 0.611 |
| Returning long COVID | 393 | 1.07 (0.19) | < 0.0001 | 32.41 | 2.92 | 2.02 | 4.22 | 0.676 |
| Persisting long COVID | 407 | 1.12 (0.18) | < 0.0001 | 37.05 | 3.06 | 2.14 | 4.40 | 0.686 |
| **19. Headache** | | | | | | | | |
| All long COVID | 536 | 0.67 (0.15) | < 0.0001 | 21.49 | 1.96 | 1.48 | 2.61 | 0.613 |
| Returning long COVID | 393 | 0.89 (0.17) | < 0.0001 | 26.77 | 2.43 | 1.74 | 3.41 | 0.664 |
| Persisting long COVID | 407 | 0.91 (0.17) | < 0.0001 | 29.17 | 2.48 | 1.78 | 3.44 | 0.663 |
| **20. Lack of appetite** | | | | | | | | |
| All long COVID | 536 | 0.32 (0.13) | 0.0118 | 6.34 | 1.38 | 1.07 | 1.77 | 0.555 |
| Returning long COVID | 393 | 0.56 (0.16) | 0.0003 | 12.99 | 1.75 | 1.29 | 2.37 | 0.618 |
| Persisting long COVID | 407 | 0.45 (0.14) | 0.0015 | 10.04 | 1.57 | 1.19 | 2.08 | 0.579 |
| **21. Sleeping more than usual** | | | | | | | | |
| All long COVID | 536 | 0.51 (0.13) | < 0.0001 | 15.98 | 1.66 | 1.30 | 2.13 | 0.580 |
| Returning long COVID | 393 | 0.61 (0.16) | 0.0001 | 14.86 | 1.84 | 1.35 | 2.50 | 0.606 |
| Persisting long COVID | 407 | 0.55 (0.15) | 0.0002 | 14.09 | 1.73 | 1.30 | 2.31 | 0.584 |
| **22. Body aches or pains** | | | | | | | | |
| All long COVID | 536 | 0.56 (0.13) | < 0.0001 | 18.78 | 1.76 | 1.36 | 2.27 | 0.609 |
| Returning long COVID | 393 | 0.78 (0.16) | < 0.0001 | 23.77 | 2.18 | 1.59 | 2.98 | 0.667 |
| Persisting long COVID | 407 | 0.76 (0.15) | < 0.0001 | 24.92 | 2.13 | 1.58 | 2.87 | 0.654 |
| **23. Weak or tired** | | | | | | | | |
| All long COVID | 536 | 0.64 (0.11) | < 0.0001 | 32.10 | 1.90 | 1.52 | 2.38 | 0.649 |
| Returning long COVID | 393 | 0.87 (0.14) | < 0.0001 | 36.55 | 2.38 | 1.80 | 3.16 | 0.710 |
| Persisting long COVID | 407 | 0.88 (0.14) | < 0.0001 | 40.73 | 2.40 | 1.83 | 3.14 | 0.710 |
| **24. Chills or shivering** | | | | | | | | |
| All long COVID | 536 | 0.07 (0.16) | 0.6713 | 0.18 | 1.07 | 0.78 | 1.48 | 0.535 |
| Returning long COVID | 393 | 0.18 (0.20) | 0.3801 | 0.77 | 1.19 | 0.80 | 1.78 | 0.572 |
| Persisting long COVID | 407 | 0.07 (0.19) | 0.6950 | 0.15 | 1.08 | 0.74 | 1.57 | 0.540 |
| **25. Felt cold** | | | | | | | | |
| All long COVID | 536 | 0.51 (0.18) | 0.0035 | 8.53 | 1.67 | 1.18 | 2.35 | 0.598 |
| Returning long COVID | 393 | 0.58 (0.21) | 0.0053 | 7.76 | 1.78 | 1.19 | 2.67 | 0.628 |
| Persisting long COVID | 407 | 0.58 (0.19) | 0.0028 | 8.95 | 1.79 | 1.22 | 2.63 | 0.621 |
| **26. Felt hot** | | | | | | | | |
| All long COVID | 536 | 0.47 (0.18) | 0.0101 | 6.61 | 1.60 | 1.12 | 2.29 | 0.573 |
| Returning long COVID | 393 | 0.77 (0.22) | 0.0004 | 12.68 | 2.16 | 1.41 | 3.31 | 0.639 |
| Persisting long COVID | 407 | 0.55 (0.20) | 0.0047 | 7.97 | 1.74 | 1.18 | 2.55 | 0.571 |
| **27. Sweating** | | | | | | | | |
| All long COVID | 536 | 0.40 (0.17) | 0.0211 | 5.32 | 1.49 | 1.06 | 2.08 | 0.551 |
| Returning long COVID | 393 | 0.63 (0.22) | 0.0036 | 8.49 | 1.89 | 1.23 | 2.89 | 0.613 |
| Persisting long COVID | 407 | 0.49 (0.20) | 0.0131 | 6.15 | 1.63 | 1.11 | 2.40 | 0.564 |
| **28. Sneezing** | | | | | | | | |
| All long COVID | 536 | 0.96 (0.17) | < 0.0001 | 33.30 | 2.60 | 1.88 | 3.60 | 0.657 |
| Returning long COVID | 393 | 1.18 (0.20) | < 0.0001 | 35.23 | 3.26 | 2.21 | 4.82 | 0.701 |
| Persisting long COVID | 407 | 1.20 (0.19) | < 0.0001 | 39.56 | 3.33 | 2.29 | 4.84 | 0.702 |
| **29. Coughing** | | | | | | | | |
| All long COVID | 536 | 0.74 (0.12) | < 0.0001 | 36.81 | 2.09 | 1.65 | 2.65 | 0.656 |
| Returning long COVID | 393 | 0.94 (0.15) | < 0.0001 | 38.96 | 2.56 | 1.91 | 3.44 | 0.699 |
| Persisting long COVID | 407 | 0.87 (0.14) | < 0.0001 | 37.68 | 2.39 | 1.81 | 3.16 | 0.686 |
| **30. Coughed up mucus or phlegm** | | | | | | | | |
| All long COVID | 536 | 0.60 (0.13) | < 0.0001 | 20.90 | 1.82 | 1.41 | 2.35 | 0.611 |
| Returning long COVID | 393 | 0.71 (0.16) | < 0.0001 | 20.36 | 2.04 | 1.49 | 2.77 | 0.636 |
| Persisting long COVID | 407 | 0.72 (0.15) | < 0.0001 | 23.09 | 2.06 | 1.54 | 2.77 | 0.636 |
| **31. How many times did you vomit?** | | | | | | | | |
| All long COVID | 536 | 0.66 (0.46) | 0.1502 | 2.07 | 1.94 | 0.79 | 4.80 | 0.534 |
| Returning long COVID | 393 | 0.92 (0.59) | 0.1177 | 2.45 | 2.52 | 0.79 | 8.01 | 0.551 |
| Persisting long COVID | 407 | 0.74 (0.50) | 0.1417 | 2.16 | 2.10 | 0.78 | 5.65 | 0.532 |
| **32. How many times did you have diarrhea?** | | | | | | | | |
| All long COVID | 536 | 1.23 (0.23) | < 0.0001 | 28.16 | 3.41 | 2.17 | 5.37 | 0.649 |
| Returning long COVID | 393 | 1.70 (0.27) | < 0.0001 | 39.24 | 5.45 | 3.21 | 9.27 | 0.709 |
| Persisting long COVID | 407 | 1.33 (0.25) | < 0.0001 | 28.61 | 3.77 | 2.32 | 6.12 | 0.660 |
| **33. Loss of smell** | | | | | | | | |
| All long COVID | 536 | 0.23 (0.25) | 0.3612 | 0.83 | 1.25 | 0.77 | 2.04 | 0.503 |
| Returning long COVID | 393 | 0.85 (0.30) | 0.0054 | 7.74 | 2.33 | 1.28 | 4.24 | 0.568 |
| Persisting long COVID | 407 | 0.69 (0.29) | 0.0172 | 5.67 | 1.99 | 1.13 | 3.50 | 0.547 |
| **34. Loss of taste** | | | | | | | | |
| All long COVID | 536 | 0.42 (0.25) | 0.1008 | 2.69 | 1.52 | 0.92 | 2.50 | 0.507 |
| Returning long COVID | 393 | 1.04 (0.31) | 0.0007 | 11.59 | 2.83 | 1.56 | 5.16 | 0.569 |
| Persisting long COVID | 407 | 0.92 (0.29) | 0.0016 | 9.94 | 2.51 | 1.42 | 4.45 | 0.550 |

Supplementary Table 2. ORs of long-COVID groups, “Specific Symptoms” long-COVID group: univariate models, FLU-PRO Plus items, average symptom severity.
AUC, area under the curve; CI, confidence interval; COVID, coronavirus disease 2019;
FLU-PRO Plus, inFLUenza Patient-Reported Outcome Plus; OR, odds ratio; SE, standard error.

| **Parameters** | ***n*** | **B (SE)** | ***p*-value** | **Wald Chi-square** | **OR** | **95% CI for OR** | | **C-index (AUC)** |
| --- | --- | --- | --- | --- | --- | --- | --- | --- |
|  |  |  |  |  |  | **Lower** | **Upper** |  |
| **1. Runny or dripping nose** | | | | | | | | |
| All long COVID | 536 | 0.79 (0.18) | < 0.0001 | 18.75 | 2.21 | 1.54 | 3.17 | 0.616 |
| Returning long COVID | 397 | 1.16 (0.24) | < 0.0001 | 23.42 | 3.17 | 1.99 | 5.07 | 0.685 |
| Persisting long COVID | 413 | 1.11 (0.22) | < 0.0001 | 26.58 | 3.04 | 1.99 | 4.64 | 0.666 |
| **2. Congested or stuffy nose** | | | | | | | | |
| All long COVID | 536 | 0.77 (0.16) | < 0.0001 | 24.55 | 2.16 | 1.59 | 2.93 | 0.619 |
| Returning long COVID | 397 | 1.08 (0.21) | < 0.0001 | 27.45 | 2.96 | 1.97 | 4.43 | 0.678 |
| Persisting long COVID | 413 | 1.23 (0.19) | < 0.0001 | 41.84 | 3.41 | 2.35 | 4.94 | 0.701 |
| **3. Sinus pressure** | | | | | | | | |
| All long COVID | 536 | 0.70 (0.16) | < 0.0001 | 19.86 | 2.01 | 1.48 | 2.73 | 0.600 |
| Returning long COVID | 397 | 1.08 (0.21) | < 0.0001 | 27.29 | 2.94 | 1.96 | 4.40 | 0.676 |
| Persisting long COVID | 413 | 1.09 (0.18) | < 0.0001 | 34.76 | 2.97 | 2.07 | 4.27 | 0.676 |
| **4. Scratchy or itchy throat** | | | | | | | | |
| All long COVID | 536 | 0.67 (0.17) | < 0.0001 | 15.39 | 1.96 | 1.40 | 2.75 | 0.586 |
| Returning long COVID | 397 | 0.88 (0.22) | < 0.0001 | 16.44 | 2.41 | 1.58 | 3.69 | 0.631 |
| Persisting long COVID | 413 | 0.97 (0.20) | < 0.0001 | 24.42 | 2.65 | 1.80 | 3.89 | 0.634 |
| **5. Sore or painful throat** | | | | | | | | |
| All long COVID | 536 | 0.43 (0.18) | 0.0195 | 5.46 | 1.53 | 1.07 | 2.19 | 0.547 |
| Returning long COVID | 397 | 0.66 (0.24) | 0.0050 | 7.87 | 1.94 | 1.22 | 3.07 | 0.604 |
| Persisting long COVID | 413 | 0.79 (0.21) | 0.0001 | 14.48 | 2.20 | 1.47 | 3.31 | 0.596 |
| **6. Difficulty swallowing** | | | | | | | | |
| All long COVID | 536 | 0.32 (0.21) | 0.1277 | 2.32 | 1.37 | 0.91 | 2.06 | 0.515 |
| Returning long COVID | 397 | 0.61 (0.28) | 0.0287 | 4.79 | 1.84 | 1.07 | 3.19 | 0.584 |
| Persisting long COVID | 413 | 0.66 (0.24) | 0.0051 | 7.85 | 1.94 | 1.22 | 3.08 | 0.549 |
| **7. Teary or watery eyes** | | | | | | | | |
| All long COVID | 536 | 0.69 (0.20) | 0.0005 | 12.02 | 1.99 | 1.35 | 2.93 | 0.576 |
| Returning long COVID | 397 | 0.98 (0.26) | 0.0002 | 13.80 | 2.66 | 1.59 | 4.46 | 0.628 |
| Persisting long COVID | 413 | 1.10 (0.24) | < 0.0001 | 21.06 | 3.01 | 1.88 | 4.83 | 0.634 |
| **8. Sore or painful eyes** | | | | | | | | |
| All long COVID | 536 | 0.61 (0.19) | 0.0016 | 9.91 | 1.84 | 1.26 | 2.69 | 0.567 |
| Returning long COVID | 397 | 0.88 (0.25) | 0.0005 | 12.27 | 2.40 | 1.47 | 3.92 | 0.623 |
| Persisting long COVID | 413 | 0.87 (0.23) | 0.0001 | 14.84 | 2.39 | 1.53 | 3.72 | 0.607 |
| **9. Eyes sensitive to light** | | | | | | | | |
| All long COVID | 536 | 0.60 (0.18) | 0.0006 | 11.69 | 1.83 | 1.29 | 2.59 | 0.562 |
| Returning long COVID | 397 | 0.97 (0.22) | < 0.0001 | 19.19 | 2.63 | 1.70 | 4.04 | 0.609 |
| Persisting long COVID | 413 | 0.93 (0.21) | < 0.0001 | 19.30 | 2.53 | 1.67 | 3.82 | 0.605 |
| **10. Trouble breathing** | | | | | | | | |
| All long COVID | 536 | 0.90 (0.17) | < 0.0001 | 26.72 | 2.47 | 1.75 | 3.48 | 0.613 |
| Returning long COVID | 397 | 1.11 (0.22) | < 0.0001 | 24.97 | 3.03 | 1.96 | 4.67 | 0.659 |
| Persisting long COVID | 413 | 1.30 (0.21) | < 0.0001 | 38.39 | 3.66 | 2.43 | 5.51 | 0.678 |
| **11. Chest congestion** | | | | | | | | |
| All long COVID | 536 | 0.94 (0.18) | < 0.0001 | 28.33 | 2.55 | 1.81 | 3.61 | 0.629 |
| Returning long COVID | 397 | 1.26 (0.22) | < 0.0001 | 31.83 | 3.54 | 2.28 | 5.49 | 0.686 |
| Persisting long COVID | 413 | 1.23 (0.21) | < 0.0001 | 35.54 | 3.42 | 2.28 | 5.12 | 0.682 |
| **12. Chest tightness** | | | | | | | | |
| All long COVID | 536 | 0.72 (0.18) | < 0.0001 | 16.33 | 2.05 | 1.45 | 2.91 | 0.591 |
| Returning long COVID | 397 | 0.90 (0.22) | < 0.0001 | 16.76 | 2.47 | 1.60 | 3.80 | 0.630 |
| Persisting long COVID | 413 | 1.08 (0.21) | < 0.0001 | 25.84 | 2.94 | 1.94 | 4.45 | 0.657 |
| **13. Dry or hacking cough** | | | | | | | | |
| All long COVID | 536 | 0.61 (0.13) | < 0.0001 | 23.40 | 1.84 | 1.44 | 2.35 | 0.618 |
| Returning long COVID | 397 | 0.67 (0.16) | < 0.0001 | 18.37 | 1.96 | 1.44 | 2.67 | 0.637 |
| Persisting long COVID | 413 | 0.80 (0.15) | < 0.0001 | 28.79 | 2.23 | 1.66 | 2.99 | 0.663 |
| **14. Wet or loose cough** | | | | | | | | |
| All long COVID | 536 | 0.87 (0.17) | < 0.0001 | 27.21 | 2.38 | 1.72 | 3.30 | 0.627 |
| Returning long COVID | 397 | 0.95 (0.21) | < 0.0001 | 19.66 | 2.57 | 1.69 | 3.91 | 0.642 |
| Persisting long COVID | 413 | 1.05 (0.20) | < 0.0001 | 27.08 | 2.85 | 1.92 | 4.22 | 0.656 |
| **15. Felt nauseous** | | | | | | | | |
| All long COVID | 536 | 0.29 (0.18) | 0.1006 | 2.70 | 1.34 | 0.95 | 1.89 | 0.560 |
| Returning long COVID | 397 | 0.38 (0.23) | 0.0911 | 2.85 | 1.47 | 0.94 | 2.29 | 0.602 |
| Persisting long COVID | 413 | 0.33 (0.21) | 0.1120 | 2.53 | 1.39 | 0.93 | 2.08 | 0.570 |
| **16. Stomach ache** | | | | | | | | |
| All long COVID | 536 | 0.63 (0.20) | 0.0018 | 9.78 | 1.87 | 1.26 | 2.76 | 0.579 |
| Returning long COVID | 397 | 0.92 (0.25) | 0.0003 | 13.12 | 2.52 | 1.53 | 4.15 | 0.637 |
| Persisting long COVID | 413 | 0.74 (0.23) | 0.0011 | 10.59 | 2.09 | 1.34 | 3.25 | 0.598 |
| **17. Felt dizzy** | | | | | | | | |
| All long COVID | 536 | 0.55 (0.16) | 0.0005 | 12.02 | 1.74 | 1.27 | 2.38 | 0.589 |
| Returning long COVID | 397 | 0.57 (0.20) | 0.0039 | 8.31 | 1.76 | 1.20 | 2.59 | 0.613 |
| Persisting long COVID | 413 | 0.81 (0.18) | < 0.0001 | 19.18 | 2.24 | 1.56 | 3.21 | 0.645 |
| **18. Head congestion** | | | | | | | | |
| All long COVID | 536 | 0.81 (0.15) | < 0.0001 | 27.92 | 2.25 | 1.67 | 3.05 | 0.621 |
| Returning long COVID | 397 | 1.18 (0.20) | < 0.0001 | 34.71 | 3.26 | 2.20 | 4.84 | 0.701 |
| Persisting long COVID | 413 | 1.27 (0.19) | < 0.0001 | 46.03 | 3.58 | 2.48 | 5.17 | 0.710 |
| **19. Headache** | | | | | | | | |
| All long COVID | 536 | 0.71 (0.14) | < 0.0001 | 25.51 | 2.04 | 1.55 | 2.69 | 0.620 |
| Returning long COVID | 397 | 0.88 (0.18) | < 0.0001 | 25.06 | 2.42 | 1.71 | 3.42 | 0.670 |
| Persisting long COVID | 413 | 1.05 (0.17) | < 0.0001 | 37.89 | 2.87 | 2.05 | 4.01 | 0.694 |
| **20. Lack of appetite** | | | | | | | | |
| All long COVID | 536 | 0.46 (0.13) | 0.0004 | 12.72 | 1.58 | 1.23 | 2.03 | 0.583 |
| Returning long COVID | 397 | 0.64 (0.16) | < 0.0001 | 15.62 | 1.91 | 1.38 | 2.62 | 0.634 |
| Persisting long COVID | 413 | 0.64 (0.15) | < 0.0001 | 18.57 | 1.90 | 1.42 | 2.54 | 0.618 |
| **21. Sleeping more than usual** | | | | | | | | |
| All long COVID | 536 | 0.59 (0.13) | < 0.0001 | 21.79 | 1.80 | 1.40 | 2.30 | 0.595 |
| Returning long COVID | 397 | 0.62 (0.16) | 0.0002 | 14.17 | 1.86 | 1.35 | 2.57 | 0.609 |
| Persisting long COVID | 413 | 0.70 (0.15) | < 0.0001 | 21.28 | 2.01 | 1.49 | 2.70 | 0.616 |
| **22. Body aches or pains** | | | | | | | | |
| All long COVID | 536 | 0.72 (0.13) | < 0.0001 | 29.82 | 2.05 | 1.59 | 2.66 | 0.639 |
| Returning long COVID | 397 | 0.85 (0.17) | < 0.0001 | 25.34 | 2.33 | 1.68 | 3.24 | 0.684 |
| Persisting long COVID | 413 | 1.02 (0.16) | < 0.0001 | 39.43 | 2.78 | 2.02 | 3.82 | 0.711 |
| **23. Weak or tired** | | | | | | | | |
| All long COVID | 536 | 0.77 (0.11) | < 0.0001 | 44.89 | 2.16 | 1.72 | 2.71 | 0.679 |
| Returning long COVID | 397 | 0.90 (0.15) | < 0.0001 | 36.17 | 2.45 | 1.83 | 3.28 | 0.721 |
| Persisting long COVID | 413 | 1.15 (0.15) | < 0.0001 | 58.70 | 3.16 | 2.36 | 4.25 | 0.774 |
| **24. Chills or shivering** | | | | | | | | |
| All long COVID | 536 | 0.20 (0.16) | 0.2242 | 1.48 | 1.22 | 0.88 | 1.69 | 0.550 |
| Returning long COVID | 397 | 0.23 (0.22) | 0.2934 | 1.10 | 1.26 | 0.82 | 1.92 | 0.578 |
| Persisting long COVID | 413 | 0.25 (0.20) | 0.2092 | 1.58 | 1.28 | 0.87 | 1.87 | 0.561 |
| **25. Felt cold** | | | | | | | | |
| All long COVID | 536 | 0.60 (0.17) | 0.0005 | 12.13 | 1.83 | 1.30 | 2.56 | 0.605 |
| Returning long COVID | 397 | 0.61 (0.21) | 0.0045 | 8.08 | 1.84 | 1.21 | 2.79 | 0.630 |
| Persisting long COVID | 413 | 0.72 (0.20) | 0.0003 | 13.21 | 2.06 | 1.40 | 3.05 | 0.640 |
| **26. Felt hot** | | | | | | | | |
| All long COVID | 536 | 0.51 (0.18) | 0.0039 | 8.34 | 1.67 | 1.18 | 2.37 | 0.579 |
| Returning long COVID | 397 | 0.82 (0.22) | 0.0002 | 14.16 | 2.26 | 1.48 | 3.46 | 0.650 |
| Persisting long COVID | 413 | 0.65 (0.20) | 0.0009 | 11.03 | 1.92 | 1.31 | 2.83 | 0.590 |
| **27. Sweating** | | | | | | | | |
| All long COVID | 536 | 0.44 (0.17) | 0.0085 | 6.93 | 1.55 | 1.12 | 2.16 | 0.558 |
| Returning long COVID | 397 | 0.63 (0.22) | 0.0037 | 8.45 | 1.87 | 1.23 | 2.86 | 0.616 |
| Persisting long COVID | 413 | 0.60 (0.20) | 0.0030 | 8.84 | 1.81 | 1.22 | 2.69 | 0.592 |
| **28. Sneezing** | | | | | | | | |
| All long COVID | 536 | 1.01 (0.16) | < 0.0001 | 38.50 | 2.76 | 2.00 | 3.80 | 0.666 |
| Returning long COVID | 397 | 1.17 (0.21) | < 0.0001 | 31.49 | 3.23 | 2.14 | 4.86 | 0.701 |
| Persisting long COVID | 413 | 1.32 (0.20) | < 0.0001 | 44.65 | 3.74 | 2.54 | 5.51 | 0.722 |
| **29. Coughing** | | | | | | | | |
| All long COVID | 536 | 0.87 (0.12) | < 0.0001 | 49.99 | 2.39 | 1.88 | 3.04 | 0.686 |
| Returning long COVID | 397 | 0.95 (0.16) | < 0.0001 | 36.10 | 2.57 | 1.89 | 3.50 | 0.705 |
| Persisting long COVID | 413 | 1.16 (0.16) | < 0.0001 | 56.25 | 3.20 | 2.36 | 4.34 | 0.749 |
| **30. Coughed up mucus or phlegm** | | | | | | | | |
| All long COVID | 536 | 0.73 (0.13) | < 0.0001 | 30.94 | 2.07 | 1.60 | 2.68 | 0.632 |
| Returning long COVID | 397 | 0.77 (0.17) | < 0.0001 | 21.47 | 2.16 | 1.56 | 2.98 | 0.640 |
| Persisting long COVID | 413 | 0.89 (0.16) | < 0.0001 | 32.01 | 2.45 | 1.79 | 3.33 | 0.664 |
| **31. How many times did you vomit?** | | | | | | | | |
| All long COVID | 536 | 0.87 (0.47) | 0.0637 | 3.44 | 2.38 | 0.95 | 5.96 | 0.538 |
| Returning long COVID | 397 | 1.03 (0.62) | 0.0978 | 2.74 | 2.79 | 0.83 | 9.43 | 0.553 |
| Persisting long COVID | 413 | 1.14 (0.54) | 0.0357 | 4.41 | 3.14 | 1.08 | 9.11 | 0.545 |
| **32. How many times did you have diarrhea?** | | | | | | | | |
| All long COVID | 536 | 1.31 (0.22) | < 0.0001 | 34.29 | 3.70 | 2.39 | 5.73 | 0.661 |
| Returning long COVID | 397 | 1.70 (0.28) | < 0.0001 | 38.08 | 5.48 | 3.19 | 9.40 | 0.723 |
| Persisting long COVID | 413 | 1.48 (0.25) | < 0.0001 | 35.24 | 4.41 | 2.70 | 7.21 | 0.684 |
| **33. Loss of smell** | | | | | | | | |
| All long COVID | 536 | 0.57 (0.25) | 0.0229 | 5.17 | 1.76 | 1.08 | 2.87 | 0.535 |
| Returning long COVID | 397 | 1.29 (0.33) | < 0.0001 | 15.33 | 3.64 | 1.91 | 6.96 | 0.613 |
| Persisting long COVID | 413 | 1.20 (0.30) | < 0.0001 | 15.55 | 3.32 | 1.83 | 6.03 | 0.595 |
| **34. Loss of taste** | | | | | | | | |
| All long COVID | 536 | 0.75 (0.25) | 0.0031 | 8.76 | 2.13 | 1.29 | 3.51 | 0.542 |
| Returning long COVID | 397 | 1.45 (0.33) | < 0.0001 | 19.46 | 4.27 | 2.24 | 8.13 | 0.614 |
| Persisting long COVID | 413 | 1.39 (0.31) | < 0.0001 | 20.68 | 4.03 | 2.21 | 7.34 | 0.596 |

Supplementary Table 3. Final model of long-COVID predictors for “All Symptoms”, Returning long-COVID subgroup. AIC, Akaike information criterion; AUC, area under the curve; BMI, body mass index; CI, confidence interval; COPD, chronic obstructive pulmonary disease; COVID, coronavirus disease 2019; DF, degree of freedom; FLU-PRO Plus, inFLUenza Patient-Reported Outcome Plus; OR, odds ratio; SE, standard error.

| **Parameters** | **B (SE)** | ***p*-value** | **Wald Chi-square** | **OR** | **95% CI for OR** | |
| --- | --- | --- | --- | --- | --- | --- |
|  |  |  |  |  | **Lower** | **Upper** |
| **FLU-PRO Plus domains** | | | | | | |
| Nose | 1.40 (0.40) | 0.0005 | 12.03 | 4.05 | 1.84 | 8.93 |
| Throat | –0.48 (0.40) | 0.2270 | 1.46 | 0.62 | 0.28 | 1.35 |
| Eyes | –0.33 (0.45) | 0.4710 | 0.52 | 0.72 | 0.30 | 1.75 |
| Chest/Respiratory | 0.60 (0.36) | 0.0961 | 2.77 | 1.82 | 0.90 | 3.66 |
| Gastrointestinal | 1.03 (0.57) | 0.0730 | 3.21 | 2.79 | 0.91 | 8.58 |
| Body/Systemic | –0.14 (0.49) | 0.7776 | 0.08 | 0.87 | 0.33 | 2.29 |
| Sense | 0.05 (0.10) | 0.6522 | 0.20 | 1.05 | 0.86 | 1.27 |
| **Demographic and clinical characteristics** | | | | | | |
| Age, years | 0.03 (0.01) | 0.0007 | 11.52 | 1.03 | 1.01 | 1.05 |
| Sex, Ref: Female | –0.34 (0.25) | 0.1697 | 1.89 | 0.71 | 0.44 | 1.16 |
| Black/African American race,  Ref: White | –0.79 (0.45) | 0.0779 | 3.11 | 0.45 | 0.19 | 1.09 |
| Other race, Ref: White | –0.94 (0.56) | 0.0925 | 2.83 | 0.39 | 0.13 | 1.17 |
| BMI, kg/m^2^ | 0.02 (0.02) | 0.1991 | 1.65 | 1.02 | 0.99 | 1.06 |
| COPD | 1.32 (0.61) | 0.0315 | 4.63 | 3.75 | 1.12 | 12.47 |
| **Model fit** | | | | | | |
| Number of observations | 393 |  |  |  |  |  |
| AIC | 459.31 |  |  |  |  |  |
| -2Log likelihood | 431.31 |  |  |  |  |  |
| R^2^, Cox-Snell,  Max-rescaled | 0.1991, 0.2717 |  |  |  |  |  |
| Score, Chi-square  (DF, *p*-value) | 80.65  (13, < 0.0001) |  |  |  |  |  |
| Wald Chi-square  (DF, *p*-value) | 64.17  (13, < 0.0001) |  |  |  |  |  |
| Hosmer-Lemeshow,  Chi-square  (DF, *p*-value) | 10.03  (8, 0.2630) |  |  |  |  |  |
| C-index (AUC) | 0.7723 |  |  |  |  |  |

S**upplementary Table 4.** Final model of long-COVID predictors for **“**All Symptom**s”**, **P**ersisting long-COVID subgroup**.** AIC, Akaike information criterion; AUC, area under the curve; BMI, body mass index; CI, confidence interval; COPD, chronic obstructive pulmonary disease; COVID, coronavirus disease 2019; DF, degree of freedom; FLU-PRO Plus, inFLUenza Patient-Reported Outcome Plus; OR, odds ratio; SE, standard error.

| **Parameters** | **B (SE)** | ***p*-value** | **Wald Chi-square** | **OR** | **95% CI for OR** | |
| --- | --- | --- | --- | --- | --- | --- |
|  |  |  |  |  | **Lower** | **Upper** |
| **FLU-PRO Plus domains** | | | | | | |
| Nose | 1.72 (0.40) | < 0.0001 | 18.32 | 5.60 | 2.54 | 12.33 |
| Throat | –0.70 (0.40) | 0.0783 | 3.10 | 0.50 | 0.23 | 1.08 |
| Eyes | 0.00 (0.39) | 0.9941 | 0.00 | 1.00 | 0.47 | 2.16 |
| Chest/Respiratory | 0.40 (0.34) | 0.2450 | 1.35 | 1.49 | 0.76 | 2.92 |
| Gastrointestinal | –0.15 (0.54) | 0.7762 | 0.08 | 0.86 | 0.29 | 2.49 |
| Body/Systemic | 0.25 (0.46) | 0.5819 | 0.30 | 1.29 | 0.52 | 3.19 |
| Sense | –0.02 (0.10) | 0.8579 | 0.03 | 0.98 | 0.81 | 1.19 |
| **Demographic and clinical characteristics** | | | | | | |
| Age, years | 0.03 (0.01) | 0.0001 | 14.94 | 1.04 | 1.02 | 1.05 |
| Sex, Ref: Female | 0.07 (0.24) | 0.7831 | 0.08 | 1.07 | 0.67 | 1.71 |
| Black/African American race,  Ref: White | –0.47 (0.41) | 0.2538 | 1.30 | 0.63 | 0.28 | 1.40 |
| Other race,  Ref: White | –1.82 (0.78) | 0.0193 | 5.47 | 0.16 | 0.03 | 0.74 |
| BMI, kg/m^2^ | 0.02 (0.02) | 0.1616 | 1.96 | 1.03 | 0.99 | 1.06 |
| COPD | 1.85 (0.58) | 0.0014 | 10.25 | 6.34 | 2.05 | 19.65 |
| **Model fit** | | | | | | |
| Number of observations | 407 |  |  |  |  |  |
| AIC | 470.15 |  |  |  |  |  |
| -2Log likelihood | 442.15 |  |  |  |  |  |
| R^2^, Cox-Snell,  Max-rescaled | 0.2242, 0.3037 |  |  |  |  |  |
| Score, Chi-square  (DF, *p*-value) | 92.92  (13, < 0.0001) |  |  |  |  |  |
| Wald Chi-square  (DF, *p*-value) | 71.27  (13, < 0.0001) |  |  |  |  |  |
| Hosmer-Lemeshow,  Chi-square  (DF, *p*-value) | 4.54  (8, 0.8052) |  |  |  |  |  |
| C-index (AUC) | 0.7802 |  |  |  |  |  |
